# LncRNA CCAT1 promotes prostate cancer cells proliferation, migration, and invasion through regulation of miR-490-3p/FRAT1 axis

**DOI:** 10.18632/aging.203300

**Published:** 2021-07-28

**Authors:** Xiaowei Cai, Yiheng Dai, Peng Gao, Guanyu Ren, Dingcai Cheng, Bo Wang, Yi Wang, Jiang Yu, Yiheng Du, Xizhi Wang, Boxin Xue

**Affiliations:** 1 Department of Urology, Second Affiliated Hospital of Soochow University, Suzhou 215004, Jiangsu, China; 2 Department of Urology, Suzhou Kowloon Hospital Shanghai Jiao Tong University School of Medicine, Suzhou 215021, Jiangsu, China; 3 Department of Urology, Changhai Hospital, Naval Medical University, Shanghai 200433, Yangpu, China; 4 Department of Urology, Taixing People's Hospital, Taixing 225400, Jiangsu, China

**Keywords:** prostate cancer, CCAT1, miR-490-3p, FRAT1, epithelial-mesenchymal transition

## Abstract

Prostate cancer (PCa) is a prevalent cancer in males, with high incidence and mortality. Recent studies have shown the crucial role of long non-coding RNA (lncRNA) in PCa. Here, we aimed to explore the functional roles and inner mechanisms of lncRNA CCAT1 in PCa cells. qRT-PCR results showed that CCAT1 was upregulated in PCa tissues and cells. Functional assays demonstrated that CCAT1 knockdown suppressed cell proliferation, migration, invasion, yet promoted apoptosis, while CCAT1 promotion showed the opposite results. We also found that CCAT1 negatively regulated miR-490-3p expression and subsequently regulated FRAT1 expression. Inhibition of miR-490-3p or up-regulation of FRAT1 reversed the suppressive effects of CCAT1 knockdown on the PCa cells. In conclusion, CCAT1 regulated FRAT1 expression through miR-490-3p and then promote the PCa cells proliferation, migration, and invasion, which reveals the oncogenic function of CCAT1 in PCa progress.

## INTRODUCTION

Prostate cancer (PCa) is one of the most commonly diagnosed tumors among males, especially in developing countries [1]. Approximately 15% of PCa cases are high risk and could be lethal [2]. Nowadays, prostate-specific antigen (PSA) is still the major applied detection index for PCa. Considering that PCa is the most curable when diagnosed at the early stage, novel biomarkers and several different cancer-screening programs are under research and development by scientists for better sensitivity and specificity [3–6]. In addition, although the success of radical treatments like prostatectomy and radiotherapy has been demonstrated in PCa, there still exist miscellaneous problems waiting to be solved, such as the identification and localization of prostate lesions [7]. To improve the survival level and quality, personalized therapies for PCa patients requires further study, which relies on a more explicit understanding of the development of prostate cancer.

Currently, long non-coding RNAs (lncRNA) have been reported to exert multifarious impacts on the cancerous process of prostate cells, ranging from the regulation of gene expression to the changes in biological behaviors [8]. LncRNAs PCA3 (prostate cancer antigen 3) is proved to contribute to prostate cancer development by promoting the proliferation and metastasis of cancer cells [9]. Some other lncRNAs, such as PCGEM1 (prostate cancer gene expression marker 1), MALAT1 (metastasis-associated lung adenocarcinoma transcript1), are also identified as potential novel biomarkers in the pathogenesis of prostate cancer [10]. These oncogenic lncRNAs, along with their implicated networks, are considered of great potential as novel biomarkers and therapeutic targets for future treatment and prevention of PCa [11].

LncRNA colon cancer associated transcript 1 (CCAT1) is first identified as an oncogene in colorectal cancer by Nissan [12]. Meanwhile, the overexpression of CCAT1 is described to contribute to tumor formation in several malignant cancers of the digestive system, including colon cancer, esophageal squamous cell carcinoma, and gastric cancer [13–16]. However, roles of CCAT1 in other cancer categories and its complex correlation with numerous cancer-related factors have not been distinct so far. In PCa, a recent research reported that CCAT1 could promote the migration and invasion of PC-3 cells [17], indicating its potential roles in PCa prediction or treatments. Besides, another study illustrated the CCAT1/miR-28-5p/DDX5 axis in PCa progression [18], which proved the regulatory roles of CCAT1 on gene expression via miRNAs.

MicroRNAs (miRNA), another group of non-coding RNAs, have been proved to interact with lncRNAs in various diseases [19]. Besides, based on relative studies, CCAT1 may exert promotion effects on oncogenesis by regulating expression levels of specific miRNAs and downstream genes [20]. In this study, through bioinformatic analysis, miR-490-3p and a proto-oncogene FRAT1 were implicated in the effecting process of CCAT1. LncRNA could influence gene expression by sponging miRNAs. This modulation of miR-490-3p has been proved in cancer development [21], and activation of FRAT1 contributes to several solid-tumor progression [22, 23]. Hence, a series of experiments were conducted to explain their interaction relationships for in-depth exploration of their functions on PCa. In addition, existing researches suggest that miR-490-3p and FRAT1 could exert impacts on the wnt/β-catenin signaling pathway to induce the epithelial-mesenchymal transition (EMT) in colorectal cancer (CRC) [24], which helped drive CRC progression and aggressiveness.

In the current study, overexpression of CCAT1 was found in PCa tissues and cells. Knockdown of CCAT1 promoted the PCa cells proliferation, migration, and invasion, while CCAT1 overexpression inhibited the cell proliferation, migration, and invasion. We also found that CCAT1 regulated FRAT1 expression by miR-490-3p, which subsequently regulate the EMT process and led to PCa progression.

## RESULTS

### LncRNA CCAT1 was highly expressed in PCa tissues and cell lines

**Figure 1:**
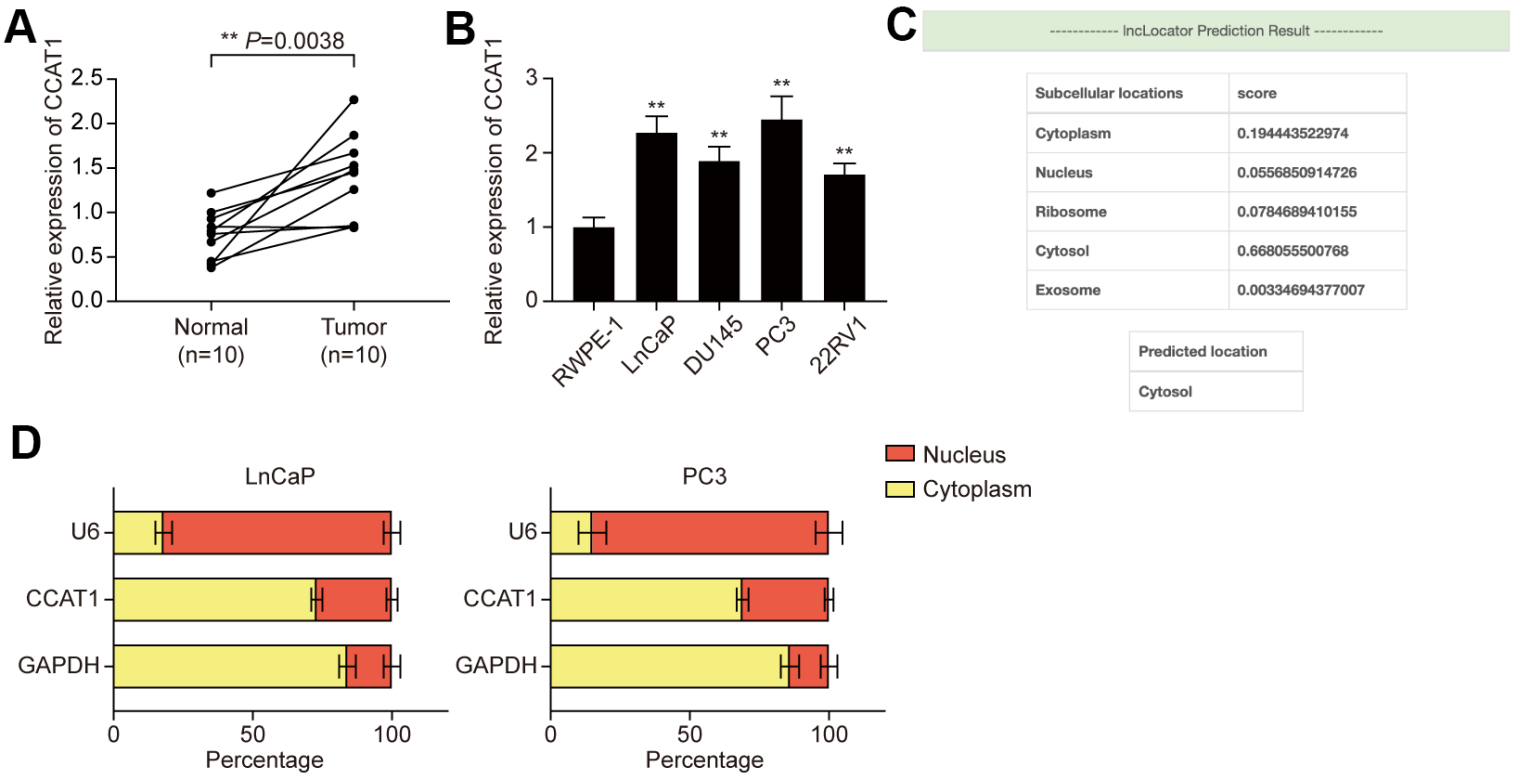
**LncRNA CCAT1 was up-regulated in PCa tissues and cell lines.** (**A**) Scatter diagram of CCAT1 expression in PCa tissues and adjacent tissues of 10 clinical samples. CCAT1 expression was generally higher in PCa tissues compared with adjacent normal tissues. (**B**) CCAT1 expression in human normal prostate epithelial cell line and four human PCa cell lines (LnCaP, DU145, PC3, and 22RV1). CCAT1 expression level was remarkably increased in PCa cell lines compared with the RWPE-1 cell line. (**C**) lncLocator (http://www.csbio.sjtu.edu.cn/bioinf/lncLocator/) prediction results of CCAT1. CCAT1 was predicted to mainly exist in the cytosol. (**D**) Subcellular location analysis of CCAT1 in LnCaP and PC3 cells. CCAT1 was distributed in the cytoplasm of LnCaP and PC3 cells. **P < 0.01, compared with the normal tissues or cells.

LncRNA CCAT1 has been reported to be overexpressed in several malignancies of the digestive system, while little is known about CCAT1 in PCa. Hence, the expression of CCAT1 in both PCa tissues and adjacent normal tissues was detected using qRT- PCR. The result showed that expression of CCAT1 was remarkably up-regulated in PCa tissues compared with adjacent normal tissues (Figure 1A). Besides, the expression of CCAT1 was also significantly up-regulated in PCa cell lines (including LnCaP, DU145, PC3, and 22RV1 cells) when compared with the normal prostate epithelial cell line RWPE-1 (Figure 1B). Given that LnCaP and PC3 showed higher CCAT1 expression, they were used for the following experiments. LncLocator was used to predict the subcellular localization of CCAT1, and the results suggested that CCAT1 was mainly located in the cytosol (Figure 1C). Subcellular fractionation further confirmed the cytoplasm location of CCAT1 in LnCaP and PC3 cells (Figure 1D).

### CCAT1 promoted cell proliferation, migration, and invasion in PCa cells

**Figure 2:**
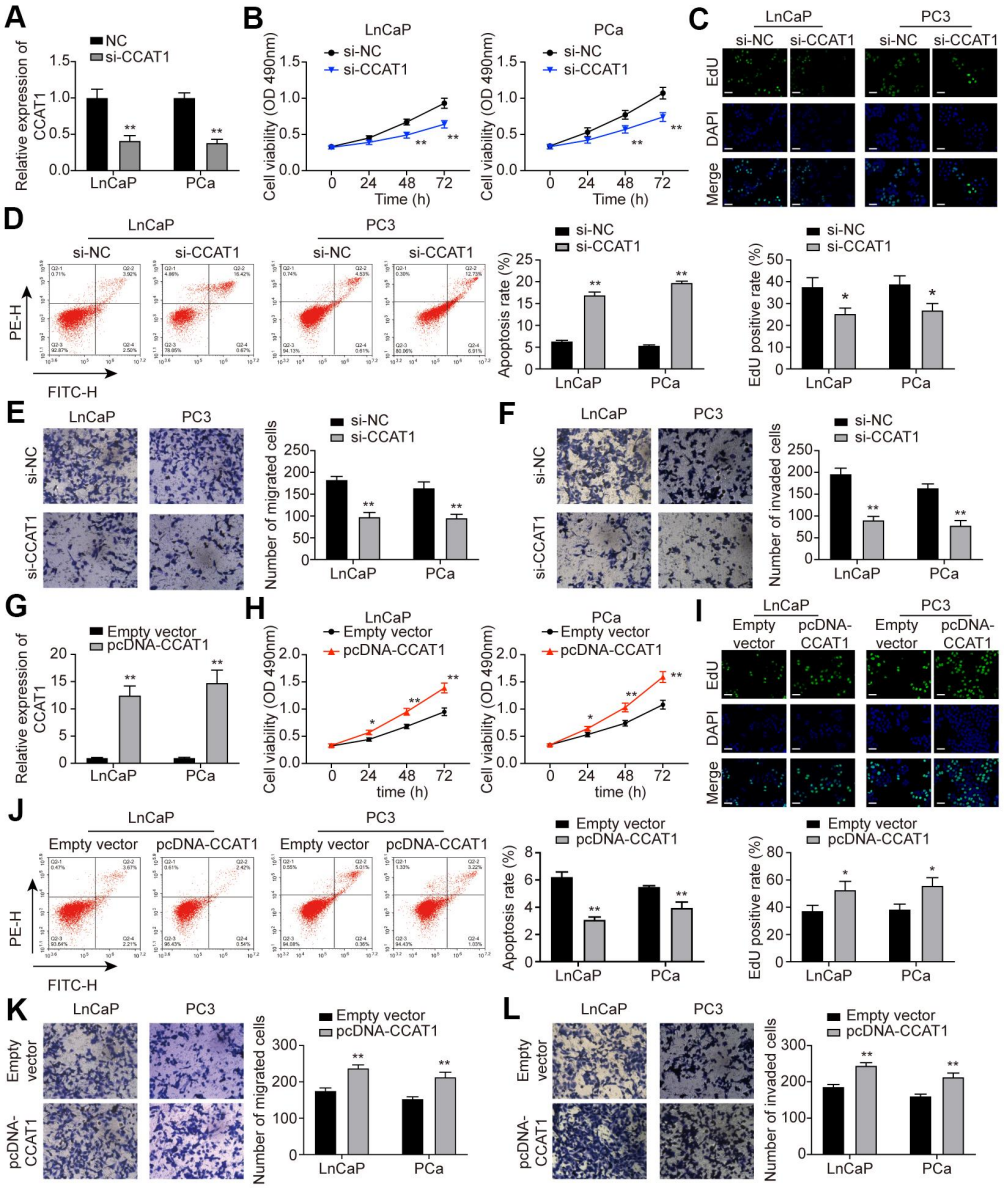
**CCAT1 promoted cell proliferation, migration, and invasion in PCa cells.** (**A**) CCAT1 expression in LnCaP and PC3 cell lines. Transfection of si-CCAT1 successfully decreased CCAT1 expression in PCa cells. (**B**) Cell viability of LnCaP and PC3 cells treated with si-NC or si-CCAT1. CCAT1 inhibition suppressed PCa cells viability. (**C**) EdU staining in LnCaP and PC3 cells treated with si-NC or si-CCAT1. CCAT1 inhibition suppressed cell proliferation in PCa cells. (**D**) Cell apoptosis of LnCaP and PC3 cells by flow cytometry detection. PCa cells with lower CCAT1 expression had higher apoptosis rate. (**E**, **F**) Cell migration and invasion of LnCaP and PC3 cells in Transwell assays. With si-CCAT1 transfection, cell migration and invasion in PCa cells were both blocked. (**G**) CCAT1 expression in LnCaP and PC3 cell lines. Transfection of pcDNA-CCAT1 successfully increased CCAT1 expression in PCa cells. (**H**) Cell viability of LnCaP and PC3 cells treated with empty vector or pcDNA-CCAT1. CCAT1 overexpression enhanced PCa cell viability. (**I**) EdU staining in LnCaP and PC3 cells treated with empty vector or pcDNA-CCAT1. CCAT1 overexpression promoted cell proliferation in PCa cells. Scale bar: 50 μm. (**J**) Cell apoptosis of LnCaP and PC3 cells by flow cytometry detection. PCa cells with higher CCAT1 expression level indicated lower apoptosis rate. (**K**, **L**) Cell migration and invasion of LnCaP and PC3 cells in Transwell assays. With pcDNA-CCAT1 transfection, cell migration and invasion in PCa cells were both enhanced. *P < 0.05, **P < 0.01, compared with the si-NC group or the empty vector group.

**Figure 3:**
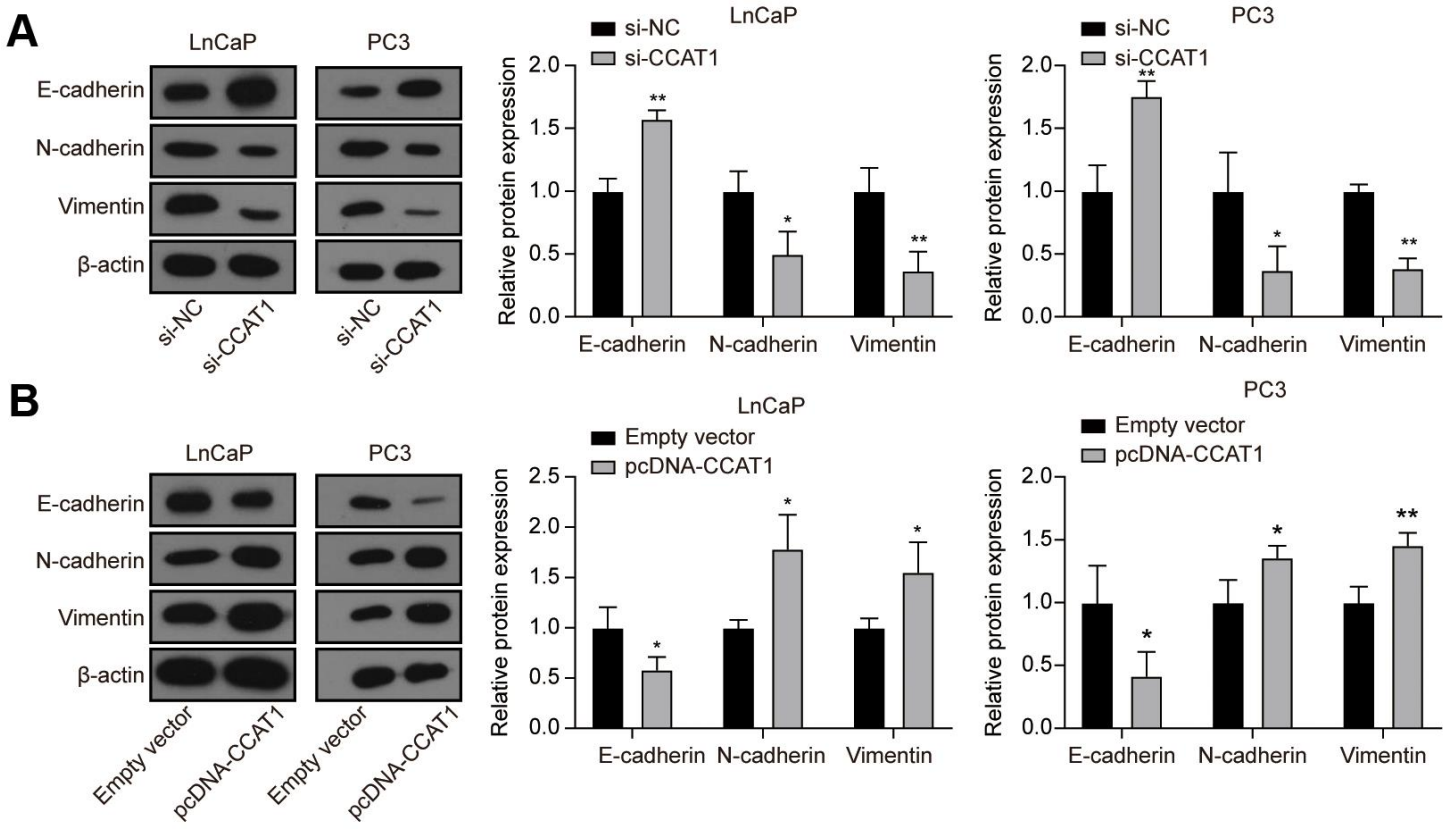
**Knockdown of CCAT1 up-regulated the expression of E-cadherin, while down-regulated the expression of N-cadherin and Vimentin.** (**A**) The protein levels of E-cadherin, N-cadherin, and Vimentin in LnCaP and PC3 cells after si-CCAT1 transfection. (**B**) The protein levels of E-cadherin, N-cadherin, and Vimentin in LnCaP and PC3 cells after pcDNA-CCAT1 transfection. *P < 0.05, **P < 0.01, compared with the si-NC group or the empty vector group.

To explore the effect of CCAT1 in PCa cells, LnCaP and PC3 cell lines were firstly transfected with si-CCAT1 to suppress the CCAT1 expression. qRT-PCR results showed that si-CCAT1 transfection greatly down-regulated the expression of CCAT1 (Figure 2A). Then, MTT assays and EdU staining analysis indicated that cell proliferation was suppressed when CCAT1 was knocked down (Figure 2B, 2C). Besides, the results of flow cytometry analysis demonstrated that the apoptosis rate of both LnCaP and PC3 cell lines was remarkably increased with the down-regulation of CCAT1 (Figure 2D). Cell migration and invasion in LnCaP and PC3 cell lines treated with si-CCAT1 were also notably decreased (Figure 2E, 2F). Therefore, knockdown of CCAT1 suppressed the malignant phenotypes of PCa cells. Conversely, CCAT1 overexpression in LnCaP and PC3 cell lines showed the opposite results. LnCaP and PC3 cell lines transfected with pcDNA-CCAT1 showed a higher CCAT1 expression (Figure 2G), resulting in enhanced cell proliferation (Figure 2H, 2I) and decreased apoptosis rate (Figure 2J). Meanwhile, cell migration and invasion were promoted with the up-regulation of CCAT1 (Figure 2K, 2L). Furthermore, as shown in Figure 3A, knockdown of CCAT1 significantly up-regulated the expression of E-cadherin, while suppressed the expression of N-cadherin and Vimentin. By contrast, overexpression of CCAT1 greatly down-regulated the expression of E-cadherin, while up-regulated the expression of N-cadherin and Vimentin (Figure 3B). Taken together, CCAT1 promoted the proliferation, migration and invasion in PCa cells through regulating the EMT process.

### MiR-490-3p was directly targeted by CCAT1 and down-regulated in PCa tissues and cell lines

**Figure 4:**
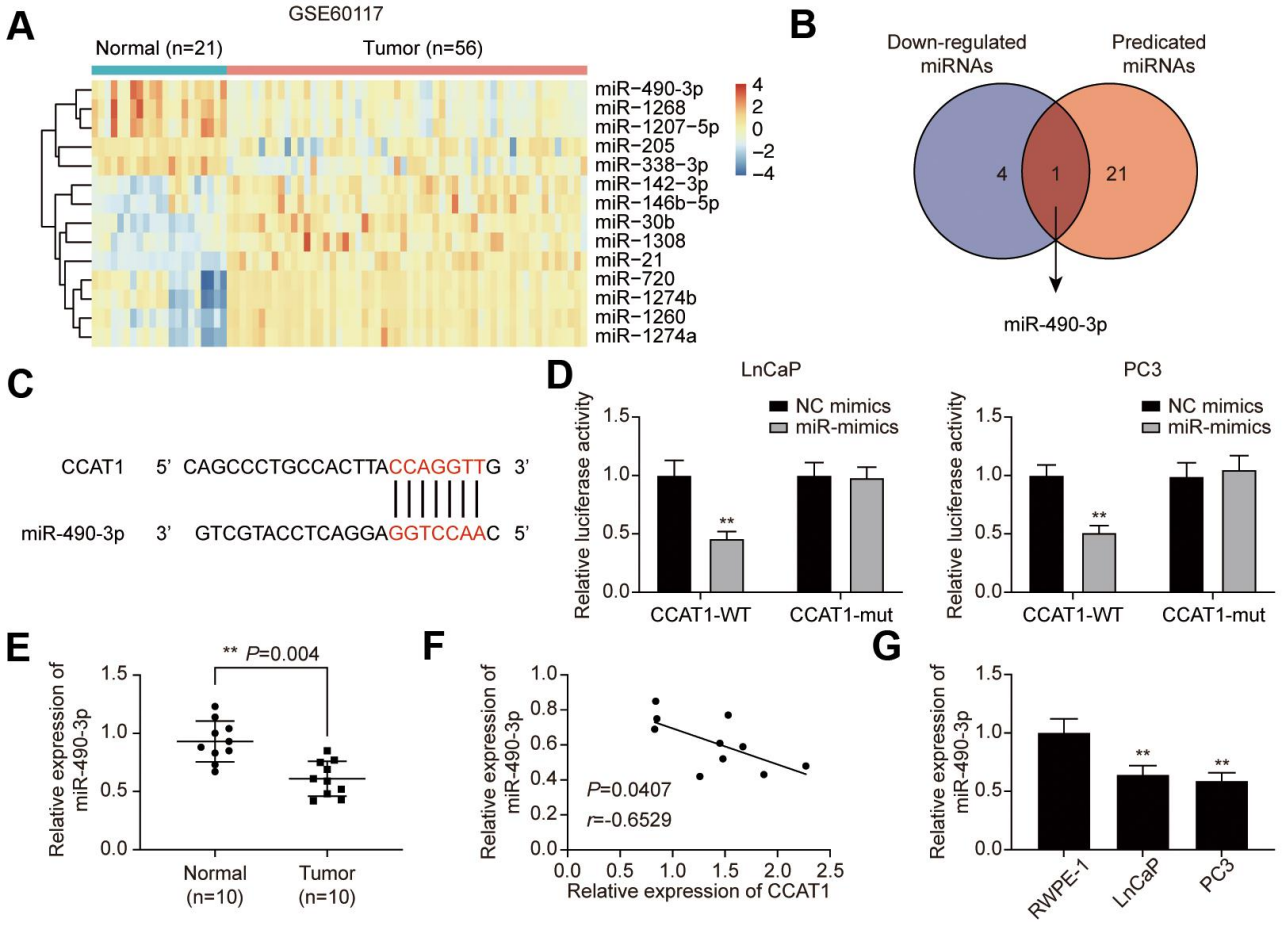
**miR-490-3p is directly targeted by CCAT1.** (**A**) Heatmap of the 14 differentially expressed miRNAs between PCa tissues and adjacent normal tissues in GSE60117. (**B**) miR-490-3p was both down-regulated in PCa and targeted by CCAT1. (**C**) Binding sites of CCAT1 and miR-490-3p. (**D**) Dual-luciferase reporter assay detection of the interaction between CCAT1 and miR-490-3p in LnCaP and PC3 cells. (**E**) miR-490-3p expression in PCa tissues and adjacent normal tissues of 10 clinical samples. miR-490-3p was decreased in PCa cells compared to that in the normal prostate tissues. (**F**) Correlation analysis of CCAT1 expression and miR-490-3p expression in PCa tissues. miR-490-3p expression was negatively correlated with CCAT1 level. (**G**) miR-490-3p expression in RWPE-1, LnCaP, and PC3 cells. miR-490-3p expression was suppressed in PCa cell lines. *P < 0.05, **P < 0.01, compared with NC mimics group or RWPE-1 group.

To explore the downstream targets of CCAT1, GSE60117 was analyzed. 14 differentially expressed miRNAs were screened out (Supplementary Table 1), and miR-490-3p was the only miRNA that predicted to be the CCAT1 target (Figure 4A, 4B). The potential binding site was illustrated in Figure 4C. Dual-luciferase reporter assay results confirmed the targeting relationship between CCAT1 and miR-490-3p in LnCaP and PC3 cell lines (Figure 4D). Besides, miR-490-3p was down-regulated in PCa tissues and cell lines, and its expression was negatively correlated with CCAT1 expression in PCa tissues (Figure 4E–4G).

### MiR-490-3p inhibited cell proliferation, migration, and invasion of PCa cells

**Figure 5:**
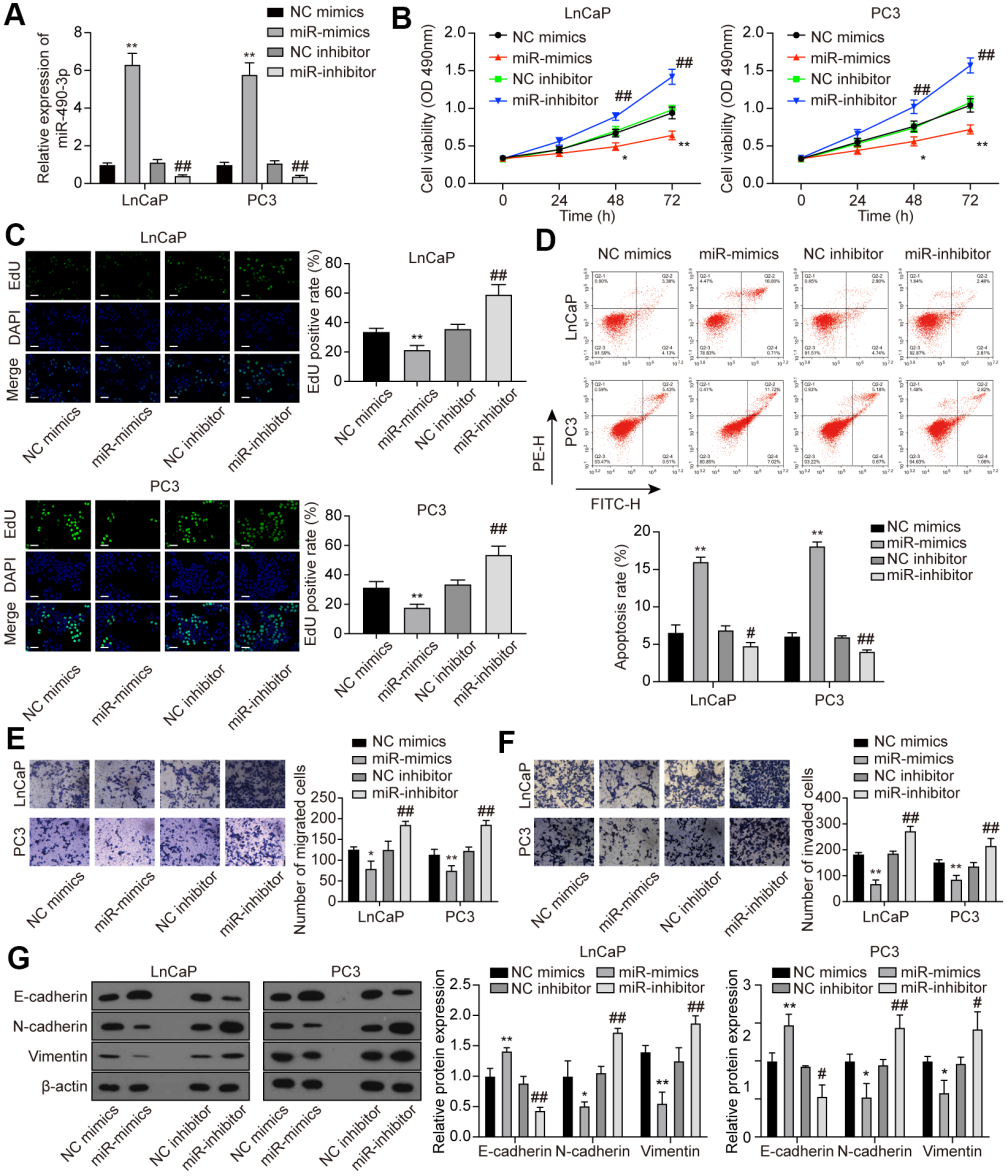
**miR-490-3p inhibited cell proliferation, migration, and invasion in PCa cells.** (**A**) Transfection efficiency of miR-490-3p mimics and inhibitor in LnCaP and PC3 cells. Compared to the NC group, PCa cells treated with miR-mimics had higher miR-490-3p expression, while those treated with miR-inhibitor had lower miR-490-3p expression. (**B**) Cell viability of LnCaP and PC3 cells with different treatments. Cell viability was suppressed in miR-mimics group and promoted in the miR-inhibitor group. (**C**) EdU staining of LnCaP and PC3 cells with different treatments. Overexpression of miR-490-3p suppressed cell proliferation, while the down-regulation of miR-490-3p promoted cell proliferation in PCa cells. Scale bar: 50 μm. (**D**) Cell apoptosis detection of LnCaP and PC3 cells with different treatments. Overexpression of miR-490-3p induced cell apoptosis in PCa cells, while the down-regulation of miR-490-3p suppressed the cell apoptosis. (**E**, **F**) Cell migration and invasion of LnCaP and PC3 cells in Transwell assays. miR-490-3p mimics treatment suppressed cell migration and invasion while the miR-490-3p inhibitor treatment activated these cell behaviors in PCa cells. (**G**) miR-490-3p overexpression up-regulated the protein expression of E-cadherin yet decreased the protein expression of N-cadherin and Vimentin, while miR-490-3p inhibition showed the opposite results. *P < 0.05, **P < 0.01, compared with the NC mimics group; #*P* < 0.05, ##*P* < 0.01, compared with the NC inhibitor group.

To explore the function of miR-490-3p in PCa cells, miR-490-3p expression was promoted or suppressed via miR-mimics or miR-inhibitor, respectively (Figure 5A). Subsequent determination of cell behaviors was respectively conducted through MTT detection, EdU staining, flow cytometry analysis, and Transwell assays. Results of above experiments demonstrated that up-regulation of miR-490-3p suppressed cell proliferation, migration, invasion, and promoted apoptosis rate in PCa cells, while miR-inhibitor transfection exerted opposite effects (Figure 5B–5F). As shown in Figure 5G, miR-490-3p overexpression up-regulated the protein expression of E-cadherin yet decreased the protein expression of N-cadherin and Vimentin, which was quite the opposite with miR-inhibitor transfection. In summary, miR-490-3p could suppress malignant cell survival in PCa.

### MiR-490-3p inhibition reversed the effects of CCAT1 suppression in PCa cells

**Figure 6:**
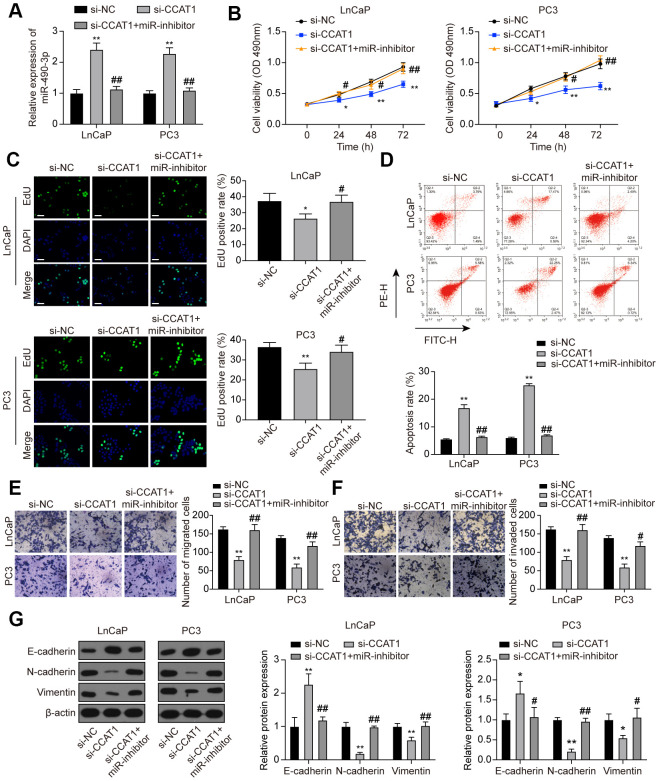
**miR-490-3p inhibition restored the influences of CCAT1 knockdown in PCa cells.** (**A**) miR-490-3p expression in LnCaP and PC3 cells transfected with si-CCAT1 or si-CCAT1+miR-inhibitor. (**B**) Cell viability of LnCaP and PC3 cells with different treatments. Cell viability was suppressed by down-regulation of CCAT1, and reversed by additional treatment with miR-inhibitor. (**C**) EdU staining results in each group of LnCaP and PC3 cells. Cell proliferation inhibition induced by si-CCAT1 was alleviated by additional treatment with miR-inhibitor. Scale bar: 50 μm. (**D**) Cell apoptosis of LnCaP and PC3 cells by flow cytometry detection. Cell apoptosis was increased by si-CCAT1 transfection and reactivated after miR-490-3p inhibition. (**E**, **F**) Cell migration and invasion of LnCaP and PC3 cells by Transwell assays. Cell migration and invasion in PCa cells were blocked in si-CCAT1 group and retrieved in si-CCAT1+miR-inhibitor group. (**G**) Down-regulation of CCAT1 increased the expression of E-cadherin yet decreased N-cadherin and Vimentin expression, and this effect was retrieved in the si-CCAT1+miR-inhibitor group. *P < 0.05, **P < 0.01, compared with the si-NC group; #*P* < 0.05, ##*P* < 0.01, compared with the si-CCAT1 group.

To investigate the interaction between CCAT1 and miR-490-3p, LnCaP and PC3 cells were transfected with si-CCAT1 or si-CCAT1+miR-inhibitor. As a result, miR-490-3p expression was notably enhanced in si-CCAT1-treated PCa cells and reversed with additional miR-inhibitor transfection (Figure 6A). MTT assays illustrated that PCa cell viability was suppressed by down-regulation of CCAT1, which was rescued to an almost normal expression level in si-CCAT1+miR-inhibitor group (Figure 6B). Similarly, the inhibition of cell proliferation induced by CCAT1 knockdown was alleviated by miR-inhibitor (Figure 6C). Flow cytometry results also indicated that the apoptosis rate of PCa cells was increased with down-regulation of CCAT1 and reactivated after miR-490-3p inhibition (Figure 6D). Cell migration and invasion in the si-CCAT1 group were both suppressed, which was retrieved in the si-CCAT1+miR-inhibitor group (Figure 6E, 6F). Furthermore, CCAT1 down-regulation increased the expression of E-cadherin yet decrease N-cadherin and Vimentin expression, which could be retrieved in the si-CCAT1+miR-inhibitor group (Figure 6G). Therefore, miR-490-3p inhibition could recover the effects of CCAT1 suppression in PCa cells.

### FRAT1 was directly targeted by miR-490-3p and up-regulated in PCa tissues and cell lines

**Figure 7:**
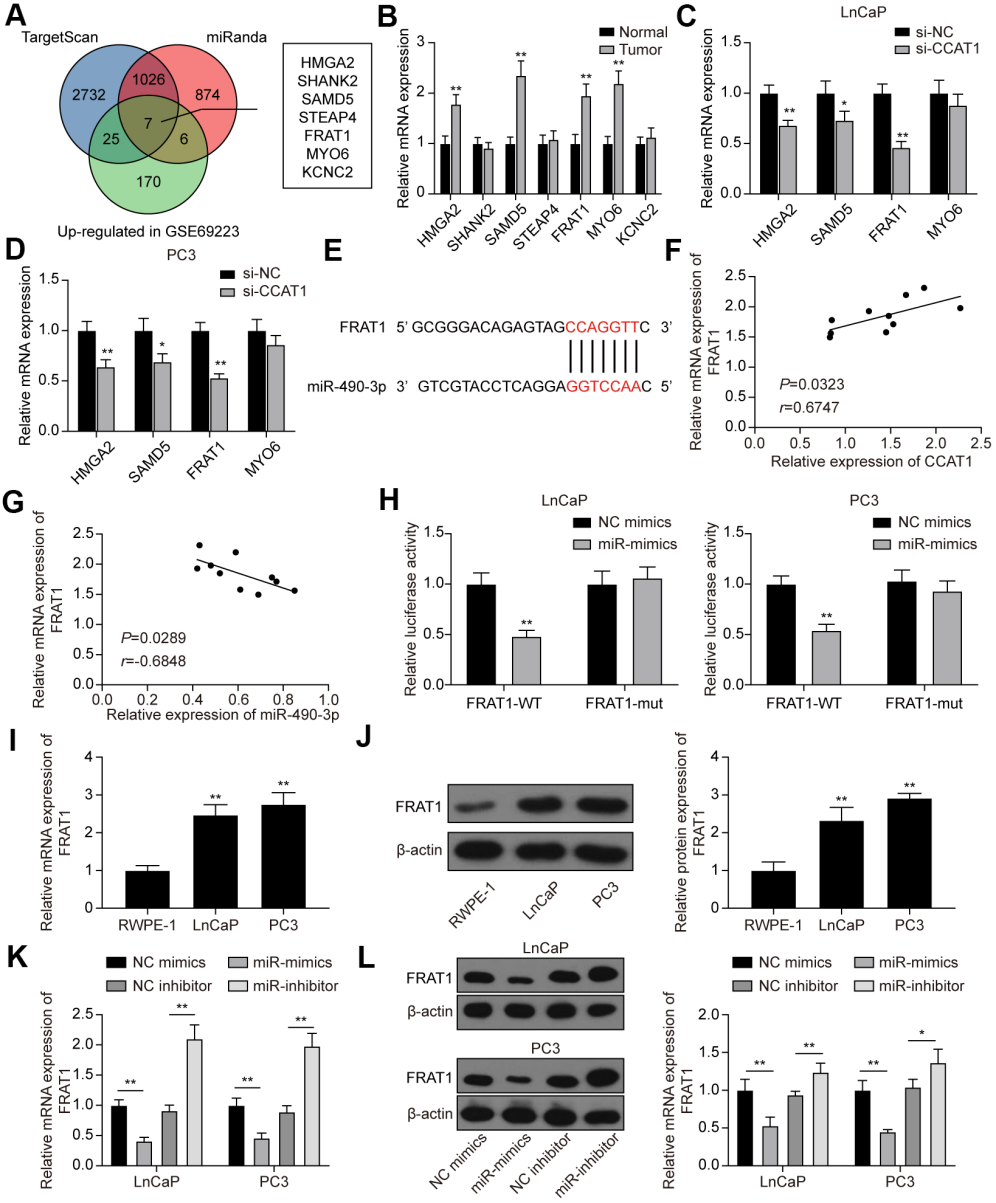
**FRAT1 was directly targeted by miR-490-3p.** (**A**) 7 potential target genes of miR-490-3p were screened out. (**B**) qRT-PCR detection of the 7 potential target genes in PCa tissues. Among them, HMGA2, SAMD5, FRAT1, and MYO6 were greatly up-regulated in PCa tissues compared with adjacent normal tissues. (**C**, **D**) Knockdown of CCAT1 greatly down-regulated the expression of HMGA2, SAMD5, and FRAT1. (**E**) Predicted binding sites between miR-490-3p and FRAT1. (**F**, **G**) FRAT1 expression was positively correlated with CCAT1 level and negatively correlated with miR-490-3p level. (**H**) Dual-luciferase reporter assay detection of the interaction between miR-490-3p and FRAT1 in LnCaP and PC3 cells. (**I**, **J**) mRNA and protein expression of FRAT1 in RWPE-1, LnCaP, and PC3 cells. Both FRAT1 mRNA and protein expression were up-regulated in PCa cell lines. (**K**, **L**) miR-490-3p overexpression greatly down-regulated the mRNA and protein expression of FRAT1, while down-regulation of miR-490-3p promoted the mRNA and protein expression of FRAT1. *P < 0.05, **P < 0.01, compared with the normal group or the si-NC group or the RWPE-1 group.

Next, we predicted target genes of miR-490-3p using TargetScan and miRanda databases. There were 3790 potential target genes predicted by TargetScan and 1913 potential target genes predicted by miRanda. Furthermore, GSE69223 was employed to analyze the differentially expressed genes in PCa, and 208 up-regulated genes were screened out (Supplementary Table 2). Combing the above results, we obtained 7 potential targets of miR-490-3p, including HMGA2, SHANK2, SAMD5, STEAP4, FRAT1, MYO6, and KCNC2 (Figure 7A). By detecting their expression in the clinical tissue samples, we found that HMGA2, SAMD5, FRAT1, and MYO6 were significantly up-regulated in PCa tissues compared with adjacent normal tissues (Figure 7B). Further analysis showed that the mRNA expression of HMGA2, SAMD5, and FRAT1 was significantly down-regulated in PCa cell lines transfected with si-CCAT1, whereas MYO6 was not affected by the CCAT1 knockdown (Figure 7C, 7D). Among the 3 significantly down-regulated genes, FRAT1 with the highest change in expression was selected as the downstream target of miR-490-3p (Figure 7E). Correlation analysis showed that FRAT1 expression was positively correlated with CCAT1 expression and negatively correlated with miR-490-3p expression (Figure 7F, 7G). Dual-luciferase reporter assay verified the targeting relationship between CCAT1 and miR-490-3p in both LnCaP and PC3 cell lines (Figure 7H). Besides, the results of qRT-PCR and western blot analysis showed that mRNA and protein expression of FRAT1 were both up-regulated in the two PCa cell lines (Figure 7I, 7J). miR-490-3p overexpression greatly down-regulated the mRNA and protein expression of FRAT1, while down-regulation of miR-490-3p resulted in the quite opposite trend (Figure 7K, 7L).

### Suppression of FRAT1 inhibited cell proliferation, migration, and invasion in PCa cells

**Figure 8:**
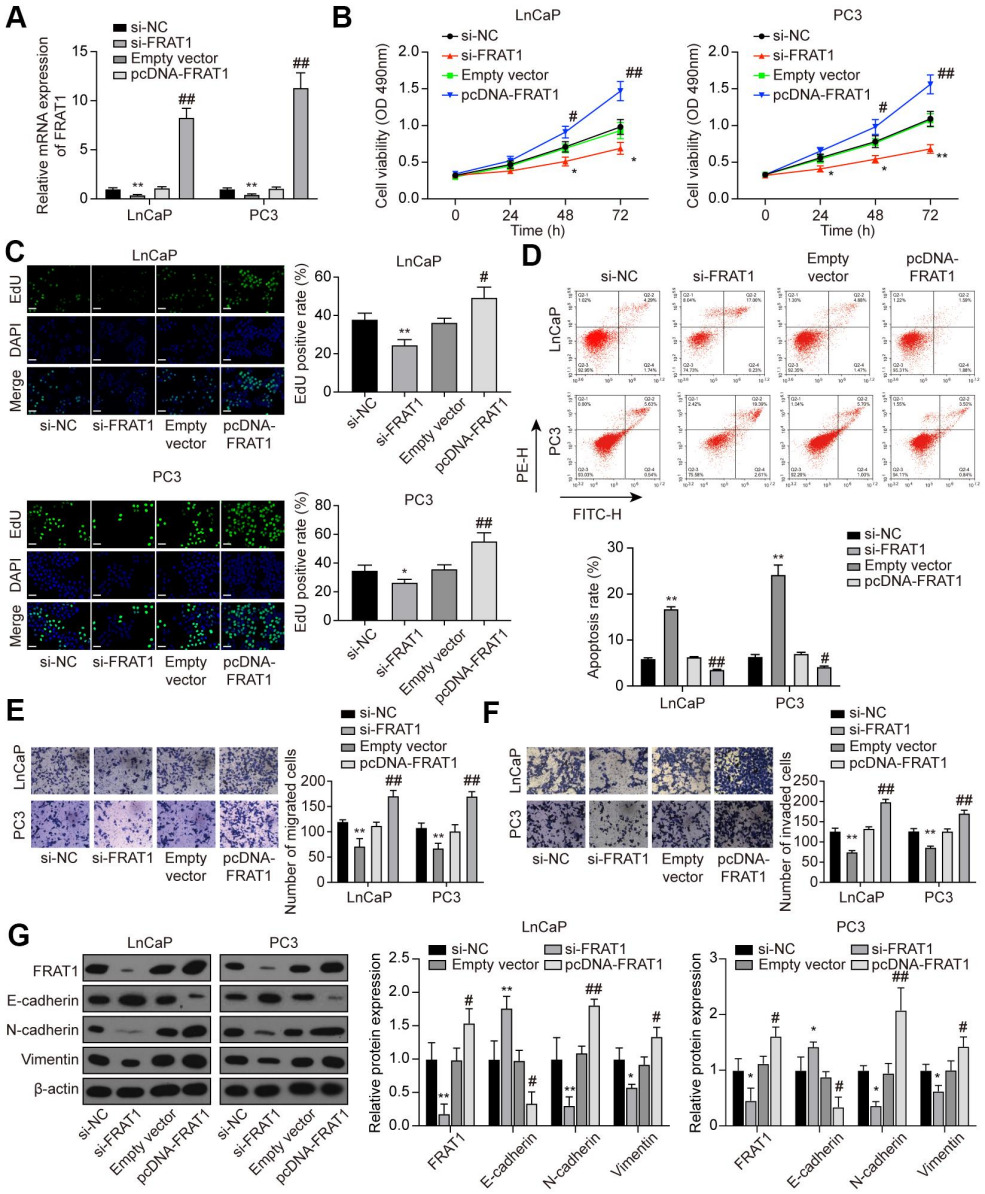
**Suppression of FRAT1 inhibited cell proliferation, migration, and invasion in PCa cells.** (**A**) Expression of FRAT1 in LnCaP and PC3 cells transfected with si-FRAT1 or pcDNA-FRAT1. (**B**) Cell viability of LnCaP and PC3 cells. Cell viability was suppressed in PCa cells treated with si-FRAT1 and promoted in PCa cells with pcDNA-FRAT1. (**C**) EdU staining results in LnCaP and PC3 cells. si-FRAT1 treatment suppressed cell proliferation while pcDNA-FRAT1 treatment promoted cell proliferation in PCa cells. Scale bar: 50 μm. (**D**) Cell apoptosis detection of LnCaP and PC3 cells by flow cytometry detection. PCa cells with lower FRAT1 expression had higher apoptosis rate, while PCa cells with higher FRAT1 expression had lower apoptosis rate. (**E**, **F**) Cell migration and invasion of LnCaP and PC3 cells in Transwell assays. Down-regulation of FRAT1 inhibited PCa cells migration and invasion, and overexpression of FRAT1 promoted PCa cells migration and invasion. (**G**) Western blot of EMT-associated protein in each group of PCa cells. E-cadherin expression was up-regulated while N-cadherin and Vimentin expression was suppressed in PCa cells transfected with si-FRAT1. PCa cells transfected with pcDNA-FRAT1 showed the opposite results. *P < 0.05, **P < 0.01, compared with the si-NC group; #*P* < 0.05, ##*P* < 0.01, compared with the empty vector group.

LnCaP and PC3 cells were treated with si-FRAT1 and pcDNA-FRAT1 to explore the effects of FRAT1 on PCa cells. qRT-PCR and western blot results showed that FRAT1 expression was suppressed after transfection with si-FRAT1, while up-regulated after pcDNA-FRAT1 transfection (Figure 8A). Further verification indicated that knockdown of FRAT1 greatly suppressed cell proliferation, migration and invasion, and promoted apoptosis in PCa cell lines (Figure 8B–8F). Conversely, up-regulation of FRAT1 greatly enhanced cell proliferation, migration, and invasion, and inhibited cell apoptosis in PCa cell lines. Western blot analysis of the EMT-related proteins showed that si-FRAT1 transfection enhanced E-cadherin expression yet declined N-cadherin and Vimentin expression in PCa cells, which was quite the opposite with pcDNA-FRAT1 transfection (Figure 8G).

### FRAT1 reversed the effects of CCAT1 suppression in PCa cells

**Figure 9:**
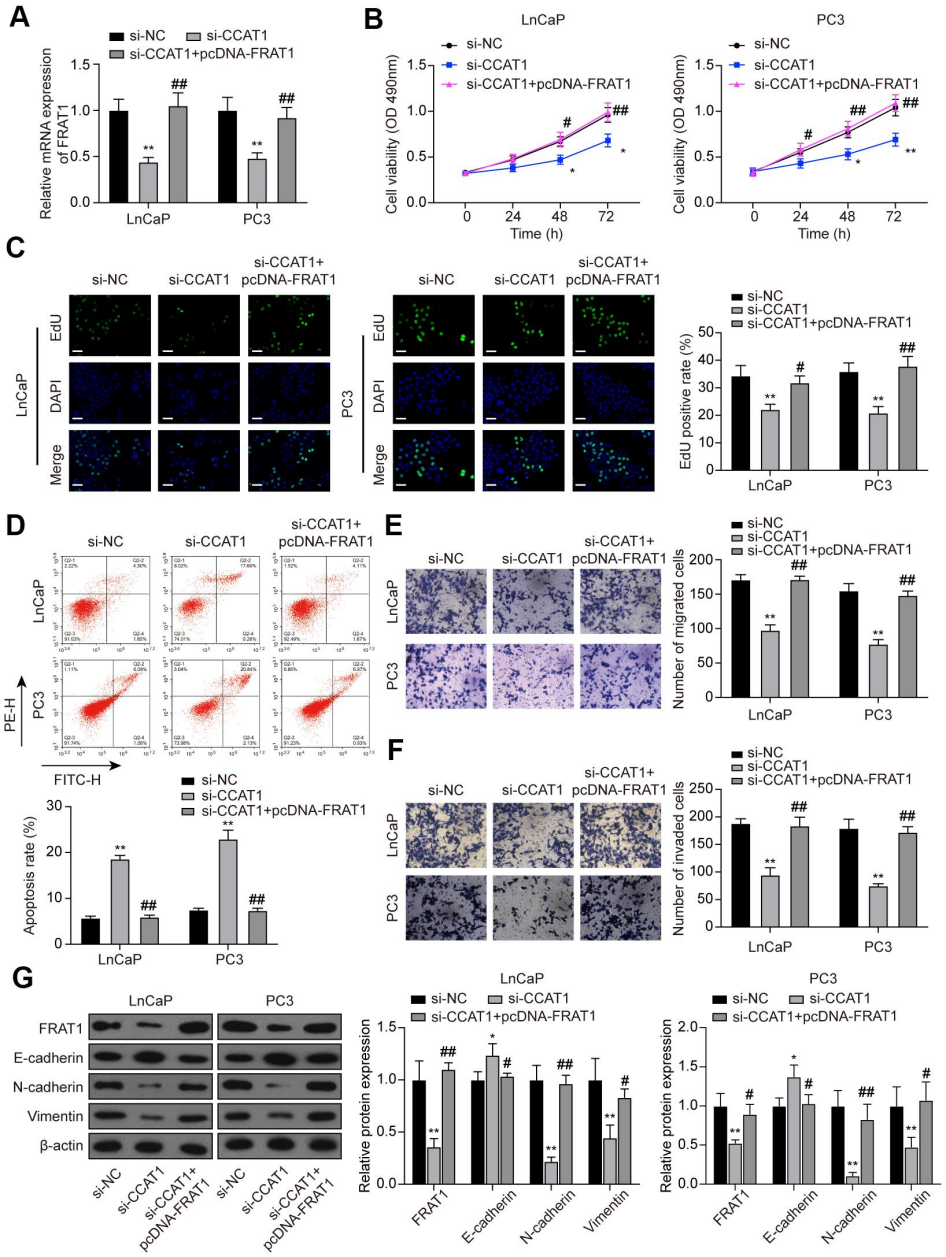
**FRAT1 overexpression reversed the effects of CCAT1 suppression in PCa cells.** (**A**) mRNA expression of FRAT1 in LnCaP and PC3 cells transfected with si-CCAT1 or si-CCAT1+pcDNA-FRAT1. (**B**) The results of MTT assays indicated that down-regulation of CCAT1 suppressed cell viability, and the suppressed ability was counteracted by pcDNA-FRAT1. (**C**) EdU staining results of LnCaP cells and PC3 cells treated with si-CCAT1 or si-CCAT1+pcDNA-FRAT1. Scale bar: 50 μm. (**D**) Flow cytometry detection of the cell apoptosis changes in LnCaP cells and PC3 cells treated with si-CCAT1 or si-CCAT1+pcDNA-FRAT1. (**E**, **F**) Cell migration and invasion were reduced with si-CCAT1 transfection, and recovered by overexpressing FRAT1. (**G**) Protein expression of FRAT1, E-cadherin, N-cadherin, and Vimentin in PCa cells were detected by western blot. *P < 0.05, **P < 0.01, compared with the si-NC group; #*P* < 0.05, ##*P* < 0.01, compared with the si-CCAT1 group.

To further explore the function of the CCAT1/miR-490-3p/FRAT1 axis in PCa, rescue assays were performed. Cells were transfected with si-CCAT1 or si-CCAT1+pcDNA-FRAT1 (Figure 9A). MTT assays and EdU staining results indicated that down-regulation of CCAT1 suppressed cell proliferation while pcDNA-FRAT1 counteracted CCAT1 inhibition-imposed effect (Figure 9B, 9C). Furthermore, the results of flow cytometry analysis demonstrated that the increased apoptosis rate caused by CCAT1 inhibition was recovered by overexpressing FRAT1 (Figure 9D). According to Transwell assay results, cell migration and invasion were notably decreased with si-CCAT1 transfection, and the suppressed ability was counteracted by pcDNA-FRAT1 (Figure 9E, 9F). Western blot analysis indicated that suppression of CCAT1 up-regulated the expression of E-cadherin and down-regulated the expression of N-cadherin and Vimentin, and FRAT1 overexpression rescued the expression changes caused by CCAT1 suppression (Figure 9G).

## DISCUSSION

PCa is the most frequent male malignancy, and its prognosis is closely related to the accuracy of diagnosis and the efficiency of treatment [25]. At present, many researchers focused on investigating the molecular mechanism during the malignant process of PCa. For instance, Yu S et al. found that LINC01116 promotes the PCa progression through regulating miR-744-5p/UBE2L3 axis [26]. Zhou Z et al. reported that ADAMTS9-AS1 inhibits the PCa progression by modulating miR-142-5p/CCND1 axis [27]. Our study identified the CCAT1/miR-490-3p/FRAT1 axis in PCa progression, which may provide a novel perspective for understanding PCa therapeutic strategy.

As mentioned before, earlier studies uncovered that CCAT1 was overexpressed in several categories of cancers [17, 28], and recent studies also reported its oncogenic effect in PCa [18, 29]. Consistently, we found that CCAT1 was up-regulated in PCa and acted as the role of oncogene in PCa cells. By inducing the EMT process, CCAT1 promoted cell proliferation, migration, and invasion in LnCaP and PC3 cell lines. In addition, CCAT1 was reported to exert suppression on several miRNAs via sponging effects in malignancies [30, 31], including PCa [18]. In this study, we identified miR-490-3p as a direct target of CCAT1 in PCa. Previous studies reported that miR-490-3p is a tumor suppressor in invasive ductal carcinoma [32], hepatocellular carcinoma [33], glioma [20], and esophageal squamous cell carcinoma [34]. Here, miR-490-3p was down-regulated in PCa tissues and cell lines, and up-regulation of miR-490-3p repressed the malignant phenotypes of PCa cells. Through rescue experiments, we further found that the inhibitory role of CCAT1 down-regulation in PCa cells progression was recovered by additional miR-490-3p inhibition. Thus, we confirmed that the CCAT1 was molecular sponge of miR-490-3p in PCa. However, considering that there were other miRNAs linked to CCAT1, these miRNAs and their downstream influences could be novel research objects in future studies for more comprehensive understanding of PCa progression.

Our results also revealed that FRAT1 was a target gene of miR-490-3p and indirectly regulated by CCAT1. Existing studies have reported the oncogenic role of FRAT1 in several cancers, such as leukemia [35, 36], cervical cancer [37], glioma [38], and meningioma [39]. Herein, we found that FRAT1 was up-regulated in PCa. *In vitro* functional assay confirmed the oncogenic role of FRAT1 in PCa. Additionally, rescue experiments suggested that FRAT1 overexpression could reverse the effects of CCAT1 suppression in PCa cells progression. Besides, FRAT1 were potent activators of Wnt/β-catenin signal transduction [40], and dysregulation of Wnt signaling contributes to the tumorigenesis by promoting the EMT process [41–43]. Thus, we further detected the EMT-related proteins in this study. The results indicated that CCAT1 could activate the EMT process through the miR-490-3p/FRAT1 axis, which could explain the promotion effects of CCAT1 on PCa cells proliferation, migration, and invasion identified in our study.

To conclude, this study provides an outline of how CCAT1 plays a positive role in PCa progression and metastasis. The regulation of CCAT1/miR-490-3p/FRAT1 axis could be applied to future PCa treatment. In addition, interactions between lncRNA CCAT1 and its target miRNAs or downstream genes are numerous and complex. Therefore, findings in this study are of certain potential and also require deeper exploration in the future.

## CONCLUSIONS

Collectively, CCAT1 and FRAT1 were up-regulated, while miR-490-3p was down-regulated in PCa tissues and cell lines. Inhibition of CCAT1 suppressed cell proliferation, migration, and invasion in PCa cells. Moreover, CCAT1 regulated FRAT1 expression by miR-490-3p, which subsequently promoted EMT processes in PCa cells. Taken together, our study may provide a novel perspective for understanding PCa therapeutic strategy.

## MATERIALS AND METHODS

### Bioinformatic analysis

Microarray data of GSE60117 and GSE69223 were downloaded from National Center for Biotechnology Information (NCBI) Gene Expression Omnibus database (GEO, https://www.ncbi.nlm.nih.gov/geo/). Differential expression analysis was performed with R software (ver 3.5.0, https://www.r-project.org/) using “limma” R package. The differentially expressed miRNAs and mRNAs were screened out with the threshold: *P* value < 0.05 (adjusted by the BH method) and FC (Fold Change) >2.

### Clinical samples

Ten localized PCa and corresponding adjacent normal prostate tissues were collected from patients at Suzhou Kowloon Hospital, from Jan 2016 to Jun 2018. All of the patients did not receive any pre-operation treatment. Written informed consent was obtained from each participant according to federal and institutional guidelines. This investigation was approved by the Hospital Ethical and Research Committee.

### Cell culture

Human normal prostate epithelial cell line RWPE-1 and 4 PCa cell lines (LnCaP, DU145, PC3, and 22RV1) were obtained from BeNa Culture Collection (Beijing, China). RWPE-1 cell was cultured in Dulbecco’s Modified Eagle Medium (DMEM, Sigma, St. Louis, MO, USA), while the PCa cell lines were cultured in RPMI 1640 medium (Sigma). Before use, the medium was supplemented with 10% fetal bovine serum (FBS, Sigma). Thereafter, five cell lines as mentioned above were separately cultured at 37° C under a humidified atmosphere of 5% CO_2_.

### qRT-PCR (Quantitative real-time polymerase chain reaction)

Total RNA from the samples was isolated with TRIzol reagent (Invitrogen, Carlsbad, CA, USA). RNA quality and concentration were confirmed using a NanoDrop 2000 (Thermo Fisher Scientific Inc., USA). After that, total RNA was reversely transcribed to cDNAs. A SuperScript^TM^ III Reverse Transcriptase Kit (Invitrogen) was applied to lncRNA/mRNA reverse transcription while the miRCURY LNA RT Kit (Qiagen, Duesseldorf, Germany) was utilized for miRNA reverse transcription. qRT-PCR was conducted with THUNDERBIRD SYBR^®^ qPCR Mix (Toyobo, Japan). Relative RNA expression was calculated with 2^-ΔΔCt^ method. GAPDH was used as the internal reference for lncRNA and mRNA and U6 was used as the internal reference for miRNA. The primer sequences were listed in Supplementary Table 3.

### Cell transfection

To confirm the roles of CCAT1 and FRAT1 in PCa cells, small interfering RNAs (siRNA) and expression vectors were designed and synthesized by GenePharma (Shanghai, China). For the determination of miR-490-3p function, miR-490-3p mimics and inhibitor were also obtained from GenePharma. All of the cell transfections were performed with Lipofectamine 2000 (Invitrogen) following the manufacturer's protocol. Detailed sequence information was supplied in Supplementary Table 4. Cell functional experiments were performed at 48h after the transfection.

### MTT assay (3-(4,5-dimethylthiazol-2-yl)-2,5-diphenyltetrazolium bromide assay)

LnCaP and PC3 cells were respectively grouped, transfected, and then maintained in 96- well plates. At 24, 48, 72 h after transfection, the cells were incubated with 10 μL MTT solution (Beyotime, Shanghai, China) at 37° C for 4 hours. Thereafter, dimethyl sulfoxide (DMSO, Beyotime) was added to dissolve the product. The absorbance at 490 nm was calculated by Microplate Reader ELx808 (BioTek Instruments, Inc., USA).

### EdU staining

An EdU Staining Proliferation Kit (Abcam, USA) was utilized to identify cell proliferation of PCa cells. Cells to be stained were firstly added with EdU solution and then incubated for 24 h under optimal growth conditions. After being washed, cells were supplemented with a fixative solution and incubated for 15 min. Subsequently, the permeabilization buffer was added for 15 min. After washed again, cells were added with reaction mix to fluorescently label EdU and incubated for 30 min. To capture the staining results, a fluorescence microscope was used to visualize cell proliferation level.

### Transwell assay

Cell migration and invasion of LnCaP and PC3 cells with different treatments were determined by using 24-well Transwell chambers (8 μm, Corning, NY, USA). Cells were maintained in serum-free medium to form suspension. For cell migration assay, the upper chambers were supplemented with 150-μL cell suspension of each group, and 500 μL DMEM medium with 10% FBS was mixed in the bottom chamber. For cell invasion assay, the upper chambers were supplemented with 150 μL cell suspension of each group and covered with Matrigel (Invitrogen), and 500 μL DMEM medium with 10% FBS was mixed in the bottom chamber. After 24 h maintenance and washing twice with PBS, the cells on the upper surface of the membrane were removed by cotton swabs. The migrated or invaded cells were counted in randomly selected fields and photographed under a microscope.

### Flow cytometry apoptosis detection analysis

PCa cells were initially maintained in 6- well plates and then received different treatments. After 48 h incubation, cells were harvested and then supplemented with the Annexin V-FITC Apoptosis Detection Kit (BD Biosciences, San Jose, CA, USA) for 15 min. Thereafter, flow cytometry analysis for cell apoptosis was conducted on the FACSCanto^TM^ II Flow Cytometer (BD Biosciences, CA, USA).

### Dual-luciferase reporter gene assay

The target prediction was performed using the TargetScan database [44] and miRanda database [45]. Then, wild-type (wt) / mutation-type (mut) CCAT1 or FRAT1 were cloned into the PmirGLO vectors (Promega, Madison, WI, USA). Thereafter, the constructed luciferase reporter gene vectors were respectively introduced into PCa cells in the presence of miR-490-3p mimics. 48 h after the transfection, relative luciferase activity was assessed with the Dual-luciferase Reporter Assay System (Promega, USA).

### Western blot analysis

Total proteins were extracted after cell transfection by RIPA lysis buffer (Beyotime) and quantified using an Enhanced BCA Protein Assay Kit (Beyotime). Thereafter, 20 μg of total protein was separated by SDS-PAGE and transferred to PVDF membranes. After that, the membranes were blocked and incubated with the following antibodies overnight at 4° C: anti-FRAT1 (ab108405), anti-E-cadherin (ab40772), anti-N-cadherin (ab76011), anti-Vimentin (ab92547) and anti-β-actin (ab8227) as the loading control. Secondary antibody (goat anti-rabbit IgG H&L (HRP), ab205718) was then added and incubated for another 1 h at room temperature. All of the mentioned antibodies were purchased from Abcam (MA, USA). Proteins were visualized by ECL-plus reagents (Millipore, Billerica, MA, USA) and the band density was measured by Image J software (Version1.48u, Bethesda, USA).

### Statistical analysis

All the experiments in this study were performed in triplicate. Data were then analyzed with GraphPad (Ver. 7.0, GraphPad Software, CA, USA) and expressed as mean ± standard deviation (SD). Correlations were analyzed by Person’s correlation coefficient. Significant differences among groups were evaluated by student’s two- tailed unpaired *t*-test or one-way ANOVA. *P* value less than 0.05 was considered statistically significant.

## Supplementary Material

Supplementary Table 1

Supplementary Table 2

Supplementary Tables 3 and 4
