# Supplementary material for: LncRNA CCAT1 promotes prostate cancer cells proliferation, migration, and invasion through regulation of miR-490-3p/FRAT1 axis
Source: Aging (Albany NY). 2021 Jul 28;13(14):18527–44. doi: 10.18632/aging.203300 (PMC8351697; doi:10.18632/aging.203300)
Supplement: Supplementary Table 1 [file aging-13-203300-s001.pdf]

SUPPLEMENTARY TABLE

Supplementary Table 1. Differentially expressed miRNAs in GSE60117.

miRNAs	log2(fold change)	P value	adjusted P value
miR-490-3p	-1.007384202	8.10×10^{-7}	8.72×10^{-7}
miR-1268	-1.742010193	4.16×10^{-11}	1.17×10^{-10}
miR-1207-5p	-1.333677981	5.98×10^{-8}	6.97×10^{-8}
miR-205	-1.054575112	0.005046033	0.025663768
miR-338-3p	-1.014914392	8.97×10^{-9}	1.57×10^{-8}
miR-142-3p	1.01799555	4.74×10^{-8}	6.64×10^{-8}
miR-146b-5p	1.05888486	5.43×10^{-8}	6.91×10^{-8}
miR-30b	1.007244991	7.20×10^{-13}	2.95×10^{-12}
miR-1308	1.250645041	3.83×10^{-9}	7.66×10^{-9}
miR-21	1.05179077	2.91×10^{-18}	4.07×10^{-17}
miR-720	1.077978783	3.76×10^{-8}	5.86×10^{-8}
miR-1274b	1.489593772	3.16×10^{-9}	7.37×10^{-9}
miR-1260	1.331635049	2.52×10^{-17}	1.76×10^{-16}
miR-1274a	1.344301474	8.43×10^{-13}	2.95×10^{-12}

Fold change: Tumor v.s. Normal.
