# Supplementary material for: LncRNA CCAT1 promotes prostate cancer cells proliferation, migration, and invasion through regulation of miR-490-3p/FRAT1 axis
Source: Aging (Albany NY). 2021 Jul 28;13(14):18527–44. doi: 10.18632/aging.203300 (PMC8351697; doi:10.18632/aging.203300)
Supplement: Supplementary Table 2 [file aging-13-203300-s002.docx]

**Supplementary Table 2. Differentially expressed genes in GSE69223**

| Gene symbol | log2(Fold Change) | P Value | adjusted P Value |
| --- | --- | --- | --- |
| BMP5 | -6.1640119 | 6.86×10^-12^ | 6.48×10^-8^ |
| PTGS1 | -4.7064003 | 2.62×10^-10^ | 3.81×10^-7^ |
| SMR3B | -4.5241471 | 7.13×10^-7^ | 7.28×10^-5^ |
| KCNJ3 | -4.4070993 | 6.06×10^-12^ | 6.48×10^-8^ |
| SMTNL2 | -4.1761404 | 1.52×10^-10^ | 3.15×10^-7^ |
| TBX5-AS1 | -4.1516083 | 1.10×10^-8^ | 3.95×10^-6^ |
| FBXL21 | -3.8229582 | 1.82×10^-7^ | 2.71×10^-5^ |
| ASPA | -3.5289026 | 2.81×10^-8^ | 7.09×10^-6^ |
| SMR3A | -3.4969491 | 3.36×10^-6^ | 0.00022529 |
| NELL2 | -3.4164198 | 2.00×10^-11^ | 9.43×10^-8^ |
| TBX5 | -3.3116431 | 1.29×10^-8^ | 4.42×10^-6^ |
| TMEM37 | -3.2940698 | 4.98×10^-9^ | 2.69×10^-6^ |
| PDE1C | -3.2052064 | 8.67×10^-6^ | 0.00041693 |
| H19 | -3.1840977 | 6.77×10^-8^ | 1.30×10^-5^ |
| CHST6 | -3.0526143 | 3.34×10^-9^ | 2.18×10^-6^ |
| TBX4 | -3.0082139 | 4.82×10^-7^ | 5.56×10^-5^ |
| CA3 | -2.9652377 | 2.03×10^-8^ | 5.79×10^-6^ |
| FOXF2 | -2.934426 | 2.45×10^-10^ | 3.81×10^-7^ |
| WIF1 | -2.7779087 | 7.73×10^-7^ | 7.73×10^-5^ |
| TSLP | -2.761426 | 3.84×10^-9^ | 2.34×10^-6^ |
| SBSPON | -2.7449393 | 3.88×10^-10^ | 4.82×10^-7^ |
| CAMK1G | -2.7330557 | 4.87×10^-7^ | 5.58×10^-5^ |
| EMX2 | -2.6201639 | 9.80×10^-5^ | 0.00235769 |
| PLAC9 | -2.5910832 | 2.46×10^-9^ | 2.02×10^-6^ |
| GPR133 | -2.5748147 | 3.52×10^-8^ | 8.12×10^-6^ |
| GPM6A | -2.5181004 | 1.85×10^-5^ | 0.00070031 |
| CFD | -2.5046456 | 2.65×10^-8^ | 6.75×10^-6^ |
| HMGCLL1 | -2.4966178 | 9.11×10^-8^ | 1.61×10^-5^ |
| LMO3 | -2.4440257 | 5.33×10^-9^ | 2.80×10^-6^ |
| PENK | -2.4212456 | 2.21×10^-6^ | 0.00017243 |
| NCAM1 | -2.4061426 | 5.92×10^-10^ | 6.58×10^-7^ |
| GATA6 | -2.3824832 | 2.87×10^-10^ | 3.87×10^-7^ |
| FXYD6 | -2.3373352 | 1.94×10^-10^ | 3.33×10^-7^ |
| ZNF536 | -2.3365792 | 5.54×10^-7^ | 6.22×10^-5^ |
| VCL | -2.326959 | 7.45×10^-7^ | 7.53×10^-5^ |
| NRK | -2.3002021 | 4.80×10^-7^ | 5.56×10^-5^ |
| CHN1 | -2.2891973 | 3.65×10^-7^ | 4.57×10^-5^ |
| RBP1 | -2.2872284 | 3.33×10^-11^ | 1.26×10^-7^ |
| SPON1 | -2.2822889 | 6.40×10^-7^ | 6.73×10^-5^ |
| SEMA3E | -2.2802318 | 2.58×10^-9^ | 2.03×10^-6^ |
| ADAMTS5 | -2.2574773 | 1.75×10^-7^ | 2.67×10^-5^ |
| STMN2 | -2.2542149 | 0.001088563 | 0.01322727 |
| FAM19A1 | -2.2523767 | 2.20×10^-6^ | 0.0001721 |
| FAM212B | -2.2457016 | 1.16×10^-6^ | 0.00010611 |
| ATRNL1 | -2.1785298 | 1.01×10^-6^ | 9.44×10^-5^ |
| RNF112 | -2.1774976 | 3.38×10^-8^ | 7.88×10^-6^ |
| FLJ30901 | -2.1701194 | 0.003213014 | 0.02886824 |
| STAC | -2.1677896 | 1.83×10^-7^ | 2.71×10^-5^ |
| C7 | -2.1586931 | 1.20×10^-9^ | 1.14×10^-6^ |
| SPOCK3 | -2.1569218 | 2.44×10^-5^ | 0.00085923 |
| NRP2 | -2.1502548 | 0.000280099 | 0.00502609 |
| S100A4 | -2.1310872 | 1.45×10^-8^ | 4.83×10^-6^ |
| PRKCB | -2.1295382 | 2.17×10^-8^ | 5.97×10^-6^ |
| PNMAL2 | -2.1152842 | 6.59×10^-6^ | 0.00035716 |
| EPHB1 | -2.102905 | 4.09×10^-5^ | 0.00125418 |
| IGF2 | -2.1015986 | 1.40×10^-9^ | 1.22×10^-6^ |
| TMIE | -2.0867417 | 5.28×10^-5^ | 0.00149025 |
| LRRN3 | -2.0781311 | 2.79×10^-7^ | 3.80×10^-5^ |
| CES1 | -2.0746181 | 7.73×10^-9^ | 3.25×10^-6^ |
| CDKL1 | -2.0715283 | 0.0004414 | 0.00701246 |
| SYT9 | -2.0644704 | 0.00029675 | 0.00525994 |
| WNT2B | -2.0560216 | 2.84×10^-5^ | 0.00096333 |
| MAP1B | -2.0161979 | 1.03×10^-8^ | 3.93×10^-6^ |
| MYH6 | -2.0012737 | 0.00152763 | 0.01690953 |
| EDNRB | -1.9951852 | 6.15×10^-6^ | 0.00034505 |
| SLC14A1 | -1.9826287 | 2.43×10^-5^ | 0.00085871 |
| SMOC1 | -1.9813418 | 1.83×10^-7^ | 2.71×10^-5^ |
| HLA-DRB4 | -1.9798694 | 0.003883538 | 0.03310623 |
| CCDC80 | -1.9684171 | 3.33×10^-8^ | 7.86×10^-6^ |
| DPT | -1.9585862 | 4.72×10^-6^ | 0.00028722 |
| IGDCC4 | -1.9568356 | 3.81×10^-6^ | 0.00024465 |
| SLC8A1 | -1.9534482 | 1.69×10^-6^ | 0.00014176 |
| SEMA3A | -1.9477418 | 0.002315016 | 0.02275239 |
| PGF | -1.9464183 | 4.25×10^-8^ | 9.14×10^-6^ |
| MASP1 | -1.9427866 | 0.000667092 | 0.00944953 |
| C1QL1 | -1.940281 | 2.05×10^-8^ | 5.79×10^-6^ |
| ANGPTL1 | -1.9353444 | 5.05×10^-6^ | 0.00029793 |
| SLITRK6 | -1.9263303 | 3.46×10^-5^ | 0.00111637 |
| C2orf88 | -1.9105931 | 8.46×10^-7^ | 8.28×10^-5^ |
| CLCA2 | -1.9077286 | 0.003640462 | 0.03151009 |
| IGF1 | -1.9069673 | 2.91×10^-8^ | 7.23×10^-6^ |
| LGR6 | -1.9021624 | 3.73×10^-9^ | 2.34×10^-6^ |
| CLIP3 | -1.8977482 | 1.11×10^-8^ | 3.95×10^-6^ |
| VCAN | -1.8946494 | 0.004025376 | 0.03408326 |
| OLFML1 | -1.8676439 | 1.81×10^-11^ | 9.43×10^-8^ |
| NDNF | -1.8644114 | 1.99×10^-7^ | 2.89×10^-5^ |
| COL14A1 | -1.846831 | 1.56×10^-7^ | 2.44×10^-5^ |
| AB074162 | -1.84247 | 0.00136967 | 0.01561854 |
| LOC100506844 | -1.8418604 | 0.001989517 | 0.02044384 |
| PPP1R1A | -1.8326512 | 3.56×10^-6^ | 0.00023544 |
| KIF7 | -1.8263952 | 4.42×10^-5^ | 0.00133181 |
| DTNA | -1.8225813 | 1.26×10^-5^ | 0.00053556 |
| CCDC85A | -1.807745 | 2.15×10^-6^ | 0.00016896 |
| KRT14 | -1.8057758 | 0.000118983 | 0.00268692 |
| PITX2 | -1.8016932 | 0.000142934 | 0.00306207 |
| HOTS | -1.7981159 | 0.000713538 | 0.00989016 |
| C1QTNF7 | -1.7972984 | 1.52×10^-7^ | 2.40×10^-5^ |
| GPSM2 | -1.7951417 | 0.004425713 | 0.03659687 |
| F10 | -1.7903984 | 1.14×10^-7^ | 1.94×10^-5^ |
| PTGFR | -1.7903877 | 0.001984243 | 0.02042063 |
| EPHA7 | -1.7863048 | 5.24×10^-5^ | 0.00148872 |
| SAMD12 | -1.7826681 | 7.62×10^-6^ | 0.00038814 |
| DCN | -1.7786222 | 1.54×10^-8^ | 4.83×10^-6^ |
| GUCA1C | -1.7675782 | 0.000664909 | 0.00944513 |
| KIAA0408 | -1.7634968 | 0.003385425 | 0.02995263 |
| PTGER2 | -1.7529338 | 6.27×10^-5^ | 0.00168634 |
| NTRK1 | -1.7468199 | 0.002265226 | 0.02242172 |
| ADRA1A | -1.744685 | 6.90×10^-7^ | 7.08×10^-5^ |
| HIF3A | -1.7361289 | 7.73×10^-6^ | 0.00039238 |
| C1S | -1.7291528 | 0.00257159 | 0.02446636 |
| EFEMP1 | -1.725041 | 3.97×10^-5^ | 0.00123726 |
| HEPH | -1.7244287 | 2.78×10^-9^ | 2.10×10^-6^ |
| FXYD1 | -1.7149826 | 4.25×10^-8^ | 9.14×10^-6^ |
| DDO | -1.7140402 | 0.000522494 | 0.00793612 |
| RTDR1 | -1.6961234 | 0.001113518 | 0.0133927 |
| PCDH10 | -1.6936346 | 1.40×10^-7^ | 2.24×10^-5^ |
| TMEM100 | -1.6921854 | 4.91×10^-7^ | 5.59×10^-5^ |
| SRRM4 | -1.6900772 | 0.001939574 | 0.02018693 |
| FGF7 | -1.6897871 | 2.70×10^-7^ | 3.74×10^-5^ |
| RASGRF1 | -1.6753066 | 0.003334804 | 0.02968023 |
| CPEB1 | -1.6706293 | 8.18×10^-6^ | 0.00040342 |
| COL13A1 | -1.6629689 | 3.00×10^-8^ | 7.36×10^-6^ |
| MMP16 | -1.6603336 | 3.20×10^-7^ | 4.14×10^-5^ |
| AF070581 | -1.6436402 | 4.61×10^-8^ | 9.80×10^-6^ |
| LAMB1 | -1.6421133 | 0.000112645 | 0.00259247 |
| CHRDL1 | -1.6342211 | 5.07×10^-5^ | 0.0014567 |
| ZEB2 | -1.6308147 | 1.53×10^-6^ | 0.00013028 |
| CLU | -1.6269508 | 3.05×10^-6^ | 0.00021355 |
| UNC5B-AS1 | -1.6218752 | 0.003139558 | 0.02834302 |
| FBLN1 | -1.6188403 | 8.55×10^-7^ | 8.32×10^-5^ |
| NAV3 | -1.615293 | 0.001597738 | 0.01750102 |
| LDB3 | -1.6111041 | 1.14×10^-5^ | 0.00050419 |
| LOC100507054 | -1.6096307 | 0.000235618 | 0.00443342 |
| KCNT2 | -1.601673 | 0.00012586 | 0.00279795 |
| ANKDD1A | -1.5984983 | 9.38×10^-7^ | 8.90×10^-5^ |
| CD200 | -1.5930244 | 2.89×10^-6^ | 0.00020729 |
| MYH11 | -1.5914297 | 0.000757313 | 0.01028715 |
| KCNMA1 | -1.5866641 | 4.74×10^-6^ | 0.00028722 |
| ITGA9 | -1.5842654 | 3.69×10^-7^ | 4.57×10^-5^ |
| SFRP2 | -1.5799021 | 1.24×10^-5^ | 0.00053126 |
| RBFOX1 | -1.5727118 | 1.18×10^-5^ | 0.00051572 |
| PDGFC | -1.5657027 | 0.000592158 | 0.00867203 |
| ST8SIA1 | -1.5656817 | 7.51×10^-5^ | 0.00194335 |
| CPNE5 | -1.5551177 | 4.22×10^-7^ | 5.11×10^-5^ |
| SEMA6D | -1.5515866 | 9.16×10^-6^ | 0.000434 |
| RRAD | -1.5502619 | 5.59×10^-6^ | 0.00032201 |
| CDH2 | -1.5479388 | 0.002188537 | 0.02190276 |
| SGCD | -1.5417638 | 6.58×10^-9^ | 3.03×10^-6^ |
| ANGPT1 | -1.5344763 | 2.81×10^-7^ | 3.80×10^-5^ |
| EHD2 | -1.5304469 | 5.99×10^-5^ | 0.00164218 |
| BDNF | -1.5304042 | 3.95×10^-5^ | 0.00123485 |
| HSD11B1 | -1.526127 | 6.79×10^-5^ | 0.00178977 |
| PALLD | -1.5225957 | 6.72×10^-6^ | 0.00035858 |
| SPAG17 | -1.521227 | 0.0049635 | 0.03982392 |
| FHOD3 | -1.5159092 | 1.12×10^-5^ | 0.00050077 |
| COL6A1 | -1.5138778 | 2.81×10^-6^ | 0.00020402 |
| FHL1 | -1.5047471 | 0.00243661 | 0.0236044 |
| FAT3 | -1.5042506 | 0.000661761 | 0.00942471 |
| RASL12 | -1.5032323 | 2.82×10^-7^ | 3.80×10^-5^ |
| LMOD1 | -1.4982177 | 5.00×10^-5^ | 0.0014511 |
| RHOJ | -1.4913439 | 3.24×10^-8^ | 7.75×10^-6^ |
| GSTM5 | -1.490224 | 6.09×10^-9^ | 2.95×10^-6^ |
| CHGB | -1.4888098 | 0.000862876 | 0.01132226 |
| SRPX | -1.4884719 | 7.05×10^-6^ | 0.00036965 |
| FERMT2 | -1.4840854 | 3.22×10^-6^ | 0.00021993 |
| PHACTR1 | -1.4807168 | 0.001214854 | 0.01427529 |
| IGSF1 | -1.4775791 | 0.000146753 | 0.00312263 |
| KLHL14 | -1.474538 | 0.00053954 | 0.00812968 |
| LEMD1-AS1 | -1.4694749 | 0.000295185 | 0.00524696 |
| LOC100128239 | -1.4653278 | 0.000150674 | 0.003181 |
| FW339973 | -1.464885 | 0.005894384 | 0.04494503 |
| MAOB | -1.4638402 | 3.30×10^-6^ | 0.00022312 |
| HAPLN1 | -1.462623 | 0.002098651 | 0.02118004 |
| SYT11 | -1.4624058 | 6.87×10^-8^ | 1.31×10^-5^ |
| TIMP3 | -1.4609903 | 1.96×10^-5^ | 0.0007334 |
| CCND2 | -1.460209 | 1.94×10^-8^ | 5.79×10^-6^ |
| KCNE3 | -1.4598901 | 3.01×10^-7^ | 3.95×10^-5^ |
| CACNA1A | -1.4506672 | 0.000276594 | 0.00497737 |
| FBLN5 | -1.4495738 | 1.53×10^-6^ | 0.00013028 |
| MSRB3 | -1.4469167 | 9.67×10^-5^ | 0.00234054 |
| GPR161 | -1.441833 | 6.28×10^-6^ | 0.00035021 |
| TMEM200B | -1.4398371 | 2.96×10^-7^ | 3.93×10^-5^ |
| PIK3R4 | -1.434357 | 0.003109157 | 0.02818979 |
| MFSD2A | -1.430231 | 0.000680956 | 0.00958053 |
| PRG2 | -1.4297716 | 0.001854897 | 0.01952551 |
| BZRAP1-AS1 | -1.423765 | 6.18×10^-7^ | 6.67×10^-5^ |
| POU3F1 | -1.4193103 | 0.000575738 | 0.00852357 |
| FGFR4 | -1.4161861 | 3.45×10^-5^ | 0.001116 |
| RBFOX3 | -1.4155116 | 0.000420623 | 0.00676036 |
| FENDRR | -1.4147028 | 5.81×10^-7^ | 6.38×10^-5^ |
| NUDT10 | -1.4142062 | 4.24×10^-7^ | 5.11×10^-5^ |
| NMUR1 | -1.4106673 | 0.004326103 | 0.03602544 |
| PRKCA | -1.4100086 | 2.01×10^-8^ | 5.79×10^-6^ |
| CX3CR1 | -1.4098197 | 9.59×10^-5^ | 0.00232327 |
| RCAN2 | -1.4088816 | 6.00×10^-5^ | 0.00164337 |
| TRPC1 | -1.4085747 | 1.00×10^-6^ | 9.35×10^-5^ |
| ADAMTS18 | -1.408051 | 3.24×10^-5^ | 0.00106514 |
| UNC5C | -1.4057176 | 5.88×10^-8^ | 1.17×10^-5^ |
| COLGALT2 | -1.395009 | 9.58×10^-10^ | 1.01×10^-6^ |
| PRKG1 | -1.3870033 | 2.31×10^-6^ | 0.00017713 |
| A1BG-AS1 | -1.3859593 | 0.000271678 | 0.00492588 |
| SPARC | -1.3838714 | 1.17×10^-6^ | 0.00010642 |
| PDE5A | -1.3814479 | 0.002093342 | 0.02114041 |
| ETV5 | -1.3787059 | 4.02×10^-8^ | 9.04×10^-6^ |
| LARGE | -1.3783878 | 5.66×10^-8^ | 1.15×10^-5^ |
| FGF2 | -1.3741587 | 1.18×10^-5^ | 0.00051438 |
| LTBP4 | -1.3725168 | 4.26×10^-8^ | 9.14×10^-6^ |
| SEBOX | -1.366955 | 0.004914456 | 0.03951192 |
| HEY1 | -1.3669194 | 1.00×10^-5^ | 0.00046181 |
| SRD5A2 | -1.363002 | 2.62×10^-6^ | 0.00019454 |
| CAV2 | -1.3621383 | 6.23×10^-5^ | 0.00168298 |
| CNRIP1 | -1.3617531 | 5.67×10^-8^ | 1.15×10^-5^ |
| SH3PXD2B | -1.359324 | 1.19×10^-10^ | 2.81×10^-7^ |
| FEZ1 | -1.3579577 | 3.63×10^-7^ | 4.57×10^-5^ |
| BST2 | -1.3563721 | 8.99×10^-6^ | 0.00042808 |
| GBP1P1 | -1.3546639 | 0.001102904 | 0.0133538 |
| S1PR3 | -1.3543518 | 1.70×10^-6^ | 0.00014176 |
| TMEM158 | -1.3531563 | 3.61×10^-6^ | 0.00023653 |
| LRRC17 | -1.3502904 | 5.38×10^-6^ | 0.00031171 |
| SYNPO2 | -1.349981 | 0.003475585 | 0.03048586 |
| DZIP1 | -1.3420643 | 6.64×10^-6^ | 0.00035716 |
| KCNJ8 | -1.3403081 | 1.39×10^-5^ | 0.00057657 |
| NPTX2 | -1.3375193 | 8.11×10^-6^ | 0.00040339 |
| FAT4 | -1.3355763 | 1.34×10^-7^ | 2.19×10^-5^ |
| CLIC6 | -1.3355027 | 1.88×10^-6^ | 0.00015286 |
| VSTM4 | -1.3349146 | 7.49×10^-9^ | 3.25×10^-6^ |
| PTGIS | -1.3315678 | 2.97×10^-6^ | 0.00021119 |
| PLEKHH2 | -1.3303003 | 0.000295855 | 0.00525392 |
| RAB37 | -1.3290233 | 1.97×10^-7^ | 2.88×10^-5^ |
| HCG11 | -1.325069 | 4.51×10^-7^ | 5.36×10^-5^ |
| BHMT2 | -1.3220611 | 2.57×10^-6^ | 0.00019441 |
| TESPA1 | -1.3215326 | 0.000999654 | 0.01247783 |
| CDC42EP3 | -1.3212838 | 4.89×10^-8^ | 1.03×10^-5^ |
| SCG5 | -1.3193699 | 2.58×10^-5^ | 0.00089228 |
| FBN1 | -1.3156888 | 3.11×10^-7^ | 4.05×10^-5^ |
| DNAJB4 | -1.3133544 | 2.65×10^-6^ | 0.00019589 |
| ITGA1 | -1.3116629 | 0.000860342 | 0.01132045 |
| JAM3 | -1.3109921 | 0.000506683 | 0.00775833 |
| LPIN1 | -1.3107728 | 2.16×10^-7^ | 3.11×10^-5^ |
| LOC100506870 | -1.3082882 | 1.65×10^-5^ | 0.00064798 |
| SYNE1 | -1.3054399 | 0.000350851 | 0.00591376 |
| DAB1 | -1.3016486 | 4.77×10^-6^ | 0.00028809 |
| LOC100506558 | -1.2999068 | 0.00024864 | 0.00463185 |
| CH25H | -1.2998036 | 0.000603104 | 0.00878617 |
| SLMAP | -1.2981406 | 1.25×10^-6^ | 0.0001124 |
| SYT1 | -1.2972399 | 0.002472154 | 0.02380803 |
| RBPMS2 | -1.2954564 | 0.000111086 | 0.00257226 |
| PRTFDC1 | -1.2930858 | 1.03×10^-6^ | 9.50×10^-5^ |
| FRZB | -1.2926901 | 1.91×10^-5^ | 0.0007204 |
| PRCD | -1.2925138 | 0.001687933 | 0.01818329 |
| GPX8 | -1.2901453 | 1.97×10^-5^ | 0.00073391 |
| DIRC3 | -1.2897608 | 0.005281515 | 0.04151174 |
| FAM92A1 | -1.2870694 | 0.000172825 | 0.00352006 |
| GLIS3 | -1.286906 | 0.00012211 | 0.00273697 |
| FGFR1 | -1.2836141 | 2.59×10^-5^ | 0.00089228 |
| TGFBR3 | -1.2825636 | 3.20×10^-9^ | 2.18×10^-6^ |
| AK021804 | -1.2811866 | 5.26×10^-5^ | 0.00149025 |
| AHNAK2 | -1.2785806 | 0.003287533 | 0.02937018 |
| NXPE3 | -1.2775798 | 3.24×10^-9^ | 2.18×10^-6^ |
| COL6A2 | -1.2773728 | 1.10×10^-7^ | 1.88×10^-5^ |
| MAMDC2 | -1.2766736 | 9.04×10^-5^ | 0.00221888 |
| CYP3A5 | -1.2758337 | 0.006274607 | 0.04708447 |
| MYLK | -1.2751179 | 6.46×10^-5^ | 0.00171957 |
| ADH1C | -1.2744302 | 4.25×10^-5^ | 0.00129179 |
| TSPAN18 | -1.2743924 | 5.17×10^-6^ | 0.00030309 |
| AKAP12 | -1.2715869 | 0.000406368 | 0.00659084 |
| ANO4 | -1.2705679 | 9.13×10^-5^ | 0.00223391 |
| C1QTNF1 | -1.2694717 | 6.37×10^-8^ | 1.25×10^-5^ |
| FRMD6 | -1.2664032 | 0.000109537 | 0.00254262 |
| PLK2 | -1.2638727 | 0.000252315 | 0.00468319 |
| EGFL6 | -1.2615977 | 0.000156856 | 0.00328945 |
| TBL1X | -1.260539 | 2.04×10^-6^ | 0.00016225 |
| FOXF1 | -1.2563773 | 0.000164282 | 0.00338877 |
| HMCN1 | -1.2540624 | 0.001035067 | 0.01282598 |
| CCDC3 | -1.2535534 | 0.000140946 | 0.00302869 |
| DPYSL2 | -1.2526162 | 3.97×10^-9^ | 2.34×10^-6^ |
| ITSN1 | -1.2512014 | 2.94×10^-6^ | 0.00020966 |
| CAV1 | -1.2500377 | 6.51×10^-5^ | 0.00172948 |
| NT5E | -1.2468113 | 1.99×10^-5^ | 0.00073934 |
| NID1 | -1.2458513 | 2.75×10^-6^ | 0.00020145 |
| GPR155 | -1.2430534 | 4.59×10^-6^ | 0.00028456 |
| MRVI1 | -1.2381273 | 3.58×10^-7^ | 4.54×10^-5^ |
| PCSK5 | -1.2365723 | 7.61×10^-9^ | 3.25×10^-6^ |
| ROBO2 | -1.2358177 | 4.97×10^-6^ | 0.00029744 |
| SCN7A | -1.2332137 | 1.05×10^-5^ | 0.00047862 |
| SORCS2 | -1.2278737 | 1.25×10^-7^ | 2.10×10^-5^ |
| ZNF423 | -1.2272003 | 1.85×10^-6^ | 0.00015218 |
| HHIP | -1.2251874 | 0.000890345 | 0.0115702 |
| DDR2 | -1.2234877 | 3.02×10^-9^ | 2.18×10^-6^ |
| NID2 | -1.2227063 | 2.29×10^-5^ | 0.00081995 |
| GSTM1 | -1.2219232 | 0.001277444 | 0.01482589 |
| SERPINE2 | -1.2210941 | 9.46×10^-6^ | 0.00044452 |
| SLC35G1 | -1.2184751 | 0.000985355 | 0.01235271 |
| QSOX1 | -1.2148903 | 3.40×10^-7^ | 4.34×10^-5^ |
| VIT | -1.2141199 | 6.30×10^-6^ | 0.00035026 |
| FZD7 | -1.2103754 | 1.29×10^-7^ | 2.13×10^-5^ |
| FILIP1 | -1.2103572 | 0.00265324 | 0.02504144 |
| CFH | -1.209925 | 0.000463259 | 0.0072762 |
| FGFR2 | -1.2085022 | 0.004778517 | 0.0386847 |
| MAP1A | -1.2081556 | 7.40×10^-6^ | 0.00037985 |
| PREX2 | -1.2076746 | 9.71×10^-5^ | 0.0023413 |
| GGTA1P | -1.2045737 | 0.000244556 | 0.00457514 |
| CELF2 | -1.2010628 | 0.004339909 | 0.03609912 |
| LOC400568 | -1.1991434 | 0.00322287 | 0.02892928 |
| C5orf34 | -1.1984106 | 0.000338073 | 0.00575172 |
| UBXN10-AS1 | -1.1982554 | 9.43×10^-5^ | 0.0022956 |
| CDC37L1 | -1.1957033 | 0.000242885 | 0.00455289 |
| HSPB7 | -1.1940464 | 1.43×10^-5^ | 0.00058835 |
| KCNMB1 | -1.1923517 | 2.88×10^-5^ | 0.00097082 |
| SCARA3 | -1.1921539 | 4.07×10^-5^ | 0.00125239 |
| RBMS3 | -1.1894712 | 1.04×10^-8^ | 3.93×10^-6^ |
| PSRC1 | -1.1884731 | 7.47×10^-6^ | 0.00038234 |
| WNT11 | -1.1851278 | 0.004214538 | 0.03529862 |
| BNIP2 | -1.184816 | 2.41×10^-5^ | 0.00085524 |
| SLC24A3 | -1.1814262 | 2.36×10^-5^ | 0.00083985 |
| CALD1 | -1.1804914 | 0.000655496 | 0.00937592 |
| NTNG1 | -1.179257 | 1.87×10^-5^ | 0.00070892 |
| MTMR11 | -1.1783714 | 0.000779158 | 0.01050834 |
| A2M | -1.1776319 | 1.56×10^-5^ | 0.0006261 |
| PPP1R3C | -1.1750257 | 0.001185829 | 0.01401266 |
| PGR | -1.174514 | 0.000251201 | 0.0046717 |
| HHIPL2 | -1.1740579 | 0.001067504 | 0.01308 |
| MBNL1-AS1 | -1.1736688 | 0.001499174 | 0.0167219 |
| EGFLAM | -1.1729176 | 1.87×10^-6^ | 0.00015218 |
| CCBE1 | -1.1710592 | 0.000817826 | 0.01088344 |
| GATM | -1.1708837 | 0.000791277 | 0.01061873 |
| RGS13 | -1.16981 | 0.002071975 | 0.02101904 |
| CFL2 | -1.1691314 | 0.002690086 | 0.02531334 |
| GSTM3 | -1.1684534 | 4.95×10^-6^ | 0.00029706 |
| SYNC | -1.1662903 | 1.46×10^-5^ | 0.00059534 |
| FILIP1L | -1.165528 | 3.49×10^-6^ | 0.0002321 |
| PEAK1 | -1.1649038 | 1.29×10^-7^ | 2.13×10^-5^ |
| ADAMTSL3 | -1.1634821 | 0.00026733 | 0.00487098 |
| ITM2A | -1.1603546 | 2.09×10^-5^ | 0.00076855 |
| GNAZ | -1.1585829 | 5.88×10^-6^ | 0.00033459 |
| TENM2 | -1.1572757 | 0.001433027 | 0.0161654 |
| AQP2 | -1.1570497 | 0.001510076 | 0.01677696 |
| LOC202181 | -1.1553031 | 1.87×10^-6^ | 0.00015218 |
| PODN | -1.1538234 | 3.63×10^-6^ | 0.0002376 |
| KATNAL1 | -1.153286 | 5.25×10^-6^ | 0.00030701 |
| FKBP7 | -1.1518036 | 0.000104188 | 0.00246387 |
| NEXN | -1.1512642 | 8.17×10^-5^ | 0.00206742 |
| MEG3 | -1.1512511 | 0.000665359 | 0.00944513 |
| LAMA2 | -1.1501632 | 7.84×10^-5^ | 0.00201086 |
| FBLIM1 | -1.149726 | 2.03×10^-6^ | 0.00016225 |
| PDGFRA | -1.1495337 | 0.000129796 | 0.00285546 |
| PRKAR2B | -1.1489251 | 0.00033343 | 0.0057197 |
| PPAP2B | -1.1481367 | 1.00×10^-5^ | 0.00046275 |
| TFPI | -1.1440324 | 7.80×10^-5^ | 0.00200217 |
| CCDC69 | -1.1429971 | 0.00010303 | 0.00244567 |
| TOX | -1.1409868 | 0.000115257 | 0.00261796 |
| MAFG-AS1 | -1.1381228 | 0.004514213 | 0.037166 |
| DKK3 | -1.1329272 | 1.67×10^-5^ | 0.00065429 |
| HLF | -1.1282704 | 0.000183894 | 0.00367303 |
| STXBP5L | -1.1280442 | 0.000348359 | 0.005877 |
| CRNN | -1.1268882 | 0.006306886 | 0.04727037 |
| COL1A2 | -1.125613 | 2.78×10^-6^ | 0.00020298 |
| GNAO1 | -1.1240166 | 2.29×10^-6^ | 0.00017697 |
| SLC38A5 | -1.1239279 | 2.11×10^-6^ | 0.00016678 |
| SLIT2 | -1.1226911 | 6.64×10^-7^ | 6.93×10^-5^ |
| DDIT4L | -1.1221022 | 5.74×10^-5^ | 0.00159262 |
| ENTPD1 | -1.1207198 | 0.000329193 | 0.00568045 |
| GIPC2 | -1.1193564 | 0.000461999 | 0.00726851 |
| MIR100HG | -1.116769 | 3.96×10^-5^ | 0.00123583 |
| ACSL4 | -1.1153302 | 0.002987322 | 0.02733436 |
| SYNPO | -1.1138854 | 2.38×10^-6^ | 0.00018138 |
| FAIM2 | -1.1122864 | 5.04×10^-5^ | 0.00145631 |
| IRAK3 | -1.1116737 | 2.32×10^-5^ | 0.00082689 |
| CCDC136 | -1.1104934 | 1.50×10^-5^ | 0.00060702 |
| CLMP | -1.1084882 | 1.11×10^-5^ | 0.00049652 |
| GJC1 | -1.1061922 | 0.000130278 | 0.002859 |
| RGN | -1.1041323 | 0.000750476 | 0.01021631 |
| ACAA2 | -1.1038831 | 0.002661644 | 0.02509569 |
| NFASC | -1.1038288 | 0.001129875 | 0.01350347 |
| COX7A1 | -1.1027117 | 0.000144122 | 0.00308054 |
| GLDN | -1.101421 | 0.005308087 | 0.04168591 |
| DNM3 | -1.1008673 | 0.004470334 | 0.03691738 |
| SNX7 | -1.0997483 | 0.000417211 | 0.00672055 |
| RABGAP1L | -1.0982659 | 0.000359542 | 0.00603375 |
| TACC1 | -1.0971096 | 4.05×10^-6^ | 0.00025744 |
| RGS22 | -1.096208 | 6.14×10^-5^ | 0.00167213 |
| GLT8D2 | -1.0952688 | 0.000608756 | 0.00886167 |
| COL16A1 | -1.0936359 | 8.79×10^-7^ | 8.47×10^-5^ |
| GNAL | -1.0930986 | 0.000122673 | 0.00274309 |
| TAGAP | -1.0882394 | 5.61×10^-5^ | 0.00156849 |
| TMEM200C | -1.0876472 | 2.87×10^-7^ | 3.84×10^-5^ |
| RECK | -1.0872648 | 5.75×10^-7^ | 6.35×10^-5^ |
| ENO2 | -1.0869402 | 0.000173069 | 0.00352006 |
| PEG3-AS1 | -1.0837737 | 4.05×10^-5^ | 0.00125092 |
| DCHS1 | -1.0820794 | 3.00×10^-5^ | 0.00100424 |
| DMPK | -1.0808599 | 0.000905839 | 0.01170761 |
| TNS1 | -1.0800326 | 4.43×10^-5^ | 0.00133376 |
| ARL10 | -1.0796816 | 1.19×10^-6^ | 0.00010773 |
| SULF1 | -1.0794593 | 0.004365745 | 0.03624374 |
| GBP1 | -1.0789314 | 0.000177175 | 0.00358046 |
| LOC100996693 | -1.0784823 | 0.006338455 | 0.04744464 |
| TBXA2R | -1.0777621 | 0.000121768 | 0.00273254 |
| SV2A | -1.0762796 | 1.01×10^-5^ | 0.00046275 |
| SORBS1 | -1.0761007 | 0.00033672 | 0.00574736 |
| IDH3A | -1.074694 | 9.13×10^-6^ | 0.00043325 |
| ENAH | -1.0742608 | 1.28×10^-5^ | 0.00054152 |
| MXRA7 | -1.0725974 | 0.000337874 | 0.00575172 |
| ADAMTS9-AS2 | -1.0716349 | 9.78×10^-6^ | 0.00045649 |
| TPST1 | -1.0716141 | 4.74×10^-6^ | 0.00028722 |
| TIMP2 | -1.0706524 | 5.36×10^-5^ | 0.00150383 |
| BDH2 | -1.0692423 | 5.24×10^-5^ | 0.00148872 |
| LMO4 | -1.0684949 | 3.04×10^-6^ | 0.00021344 |
| ANO6 | -1.0661818 | 0.000108259 | 0.00252849 |
| PCDH18 | -1.065712 | 1.70×10^-6^ | 0.00014176 |
| LOC100506403 | -1.0656334 | 0.004581438 | 0.03750705 |
| TPSAB1 | -1.0643739 | 0.004962373 | 0.03982392 |
| PAPPA | -1.0634496 | 1.73×10^-6^ | 0.0001424 |
| GFRA1 | -1.0617489 | 3.64×10^-5^ | 0.00116346 |
| TGFB2 | -1.0604799 | 0.000131968 | 0.00288604 |
| LAMA4 | -1.0603966 | 0.000122515 | 0.00274281 |
| RPP30 | -1.0603359 | 0.002243582 | 0.02228228 |
| ADAM33 | -1.0571306 | 0.003090157 | 0.02804444 |
| C12orf75 | -1.0554526 | 8.18×10^-5^ | 0.00206742 |
| ITIH5 | -1.0551942 | 1.23×10^-5^ | 0.00052738 |
| GBP2 | -1.0551464 | 5.70×10^-5^ | 0.0015874 |
| DAAM2 | -1.0543278 | 7.52×10^-7^ | 7.56×10^-5^ |
| NAV1 | -1.0542807 | 8.89×10^-8^ | 1.58×10^-5^ |
| TXNL1 | -1.0522492 | 2.60×10^-5^ | 0.00089621 |
| ELN | -1.0510404 | 0.000853311 | 0.01124359 |
| NR2F6 | -1.0494622 | 0.001730714 | 0.01847562 |
| IFI44L | -1.0491884 | 0.006124241 | 0.04630554 |
| GLI2 | -1.0485807 | 4.25×10^-7^ | 5.11×10^-5^ |
| COL4A4 | -1.0478143 | 0.00013578 | 0.00295231 |
| PRELP | -1.0465209 | 0.0001044 | 0.0024658 |
| SVIL | -1.0437957 | 0.000215073 | 0.00413407 |
| SCRG1 | -1.0434219 | 0.002724363 | 0.02553415 |
| KLK15 | -1.0411004 | 0.003599239 | 0.03123915 |
| PRICKLE2 | -1.0406513 | 8.28×10^-6^ | 0.00040438 |
| ZNF804A | -1.0405993 | 0.000776278 | 0.01047717 |
| PNMA1 | -1.0394804 | 0.000157623 | 0.00330188 |
| CCDC88A | -1.0390643 | 0.001492497 | 0.01667695 |
| PLN | -1.038755 | 0.001655655 | 0.01794814 |
| LPAR1 | -1.038018 | 1.13×10^-5^ | 0.00050077 |
| LOC283070 | -1.0368807 | 0.000113328 | 0.00259933 |
| KANK2 | -1.0332582 | 1.90×10^-5^ | 0.00071778 |
| PARVA | -1.032206 | 0.000111867 | 0.00258402 |
| SACS | -1.0276093 | 5.68×10^-5^ | 0.00158626 |
| CAMK4 | -1.026377 | 0.001121793 | 0.01345795 |
| TVP23A | -1.0254226 | 1.93×10^-5^ | 0.00072326 |
| TLE4 | -1.0246434 | 2.10×10^-6^ | 0.00016632 |
| LILRA2 | -1.0238701 | 0.003877836 | 0.03309472 |
| MIR143HG | -1.0223131 | 0.003782541 | 0.03251405 |
| LOC728061 | -1.0219544 | 0.000420756 | 0.00676036 |
| G0S2 | -1.0215373 | 4.64×10^-5^ | 0.00138045 |
| TPSB2 | -1.0181002 | 0.004488598 | 0.03701967 |
| SGCB | -1.0158074 | 1.63×10^-5^ | 0.00064399 |
| HTR2A | -1.0153936 | 0.000251645 | 0.00467536 |
| TRPC4 | -1.0133414 | 0.003364432 | 0.02981752 |
| GEM | -1.0126797 | 0.001588828 | 0.01742362 |
| DES | -1.012342 | 0.00017962 | 0.00361056 |
| TNC | -1.0063281 | 0.000473682 | 0.007403 |
| NT5DC2 | -1.0030797 | 2.36×10^-5^ | 0.00083855 |
| TMEM176A | -1.0015842 | 0.000331327 | 0.00570096 |
| PMP22 | -1.0005242 | 3.46×10^-5^ | 0.00111637 |
| ST14 | -1.0000261 | 0.004012039 | 0.03404018 |
| NECAB2 | 1.00109078 | 0.004885067 | 0.03935996 |
| APRT | 1.00243611 | 2.99×10^-6^ | 0.00021144 |
| GMDS-AS1 | 1.00800767 | 1.38×10^-5^ | 0.00057637 |
| LOC80154 | 1.00860544 | 0.000114285 | 0.00261114 |
| ENTPD5 | 1.01102844 | 0.000723217 | 0.00997458 |
| REPS2 | 1.01346811 | 0.000861836 | 0.01132226 |
| ABHD2 | 1.02331333 | 0.000365325 | 0.00610868 |
| AGMAT | 1.02480122 | 2.11×10^-5^ | 0.00076935 |
| ZBED9 | 1.02767222 | 0.000814196 | 0.01084925 |
| C5orf30 | 1.03022167 | 0.000909781 | 0.01172167 |
| SHROOM1 | 1.03647222 | 0.001072617 | 0.01309244 |
| BACE2 | 1.03795122 | 7.10×10^-5^ | 0.00185167 |
| BSPRY | 1.03894011 | 3.78×10^-5^ | 0.00119058 |
| ZNF511 | 1.03909133 | 7.85×10^-7^ | 7.73×10^-5^ |
| ESRRG | 1.03962478 | 0.000267155 | 0.00487098 |
| ERVH-6 | 1.04812545 | 0.005029487 | 0.04005336 |
| LENG9 | 1.06352078 | 3.34×10^-6^ | 0.00022444 |
| SAMD13 | 1.064057 | 0.000521291 | 0.00792421 |
| KIAA1549 | 1.06805867 | 3.00×10^-5^ | 0.00100424 |
| ITPR3 | 1.06924222 | 8.10×10^-6^ | 0.00040339 |
| PVRL3 | 1.06954544 | 0.001696199 | 0.01823229 |
| SERHL2 | 1.07179545 | 0.001143737 | 0.01362605 |
| TSTA3 | 1.07183133 | 1.83×10^-7^ | 2.71×10^-5^ |
| CCNB2 | 1.07244967 | 0.000151502 | 0.00319133 |
| SNHG9 | 1.07592144 | 0.000278206 | 0.00500162 |
| CDK19 | 1.076952 | 0.002223328 | 0.02214538 |
| PIEZO1 | 1.07867622 | 2.30×10^-6^ | 0.00017713 |
| RPL22L1 | 1.08414967 | 0.001260189 | 0.01470014 |
| FRMD1 | 1.08637167 | 0.000378631 | 0.0062547 |
| RPS6KC1 | 1.087642 | 0.000498213 | 0.00767439 |
| LRRC29 | 1.08764745 | 0.00131653 | 0.0151405 |
| KIAA1244 | 1.08942333 | 0.00154634 | 0.01705088 |
| RORC | 1.08946733 | 3.70×10^-7^ | 4.57×10^-5^ |
| PPP1R14B | 1.08996056 | 3.92×10^-6^ | 0.00025086 |
| SHANK2 | 1.09012689 | 0.002521909 | 0.02417629 |
| FKBP11 | 1.09081822 | 0.000659128 | 0.00941363 |
| TDRKH | 1.09355167 | 6.41×10^-5^ | 0.00171035 |
| ODC1 | 1.095267 | 6.85×10^-5^ | 0.00180221 |
| NSUN7 | 1.09608256 | 7.74×10^-5^ | 0.00199306 |
| HS3ST1 | 1.10129633 | 0.004975808 | 0.03985498 |
| ARHGEF38 | 1.10153367 | 1.19×10^-5^ | 0.00051603 |
| FKBP1A | 1.10225167 | 7.57×10^-5^ | 0.00195249 |
| MAS1L | 1.11041322 | 0.006653754 | 0.04907521 |
| CSNK1G3 | 1.11491589 | 0.001207795 | 0.01422773 |
| ELMO3 | 1.11514289 | 1.45×10^-6^ | 0.00012567 |
| SEL1L3 | 1.11633689 | 3.68×10^-5^ | 0.00117239 |
| ARHGEF26 | 1.11650356 | 0.000405849 | 0.00658807 |
| HOXC4 | 1.12198189 | 0.001290026 | 0.01491741 |
| GGT1 | 1.12456322 | 0.002769076 | 0.02586342 |
| SERHL | 1.125685 | 0.006347931 | 0.04744464 |
| TC2N | 1.12826367 | 0.000109357 | 0.00254157 |
| FAM189A2 | 1.12982722 | 1.30×10^-5^ | 0.00055075 |
| MBOAT2 | 1.131551 | 0.001083094 | 0.01317776 |
| NKX3-1 | 1.13510011 | 0.000282897 | 0.00506187 |
| DUS1L | 1.135204 | 1.00×10^-7^ | 1.74×10^-5^ |
| AK125860 | 1.13665967 | 0.002498897 | 0.02402883 |
| HGD | 1.13686189 | 0.006077618 | 0.0460821 |
| SLC12A8 | 1.138807 | 5.86×10^-6^ | 0.00033424 |
| ATRIP | 1.14080611 | 6.53×10^-6^ | 0.00035716 |
| GNG4 | 1.14359878 | 0.005371243 | 0.04207697 |
| FLJ20021 | 1.14651067 | 7.80×10^-7^ | 7.73×10^-5^ |
| GDPD1 | 1.14935522 | 2.49×10^-5^ | 0.00086981 |
| RP11-465B22.8 | 1.15230144 | 0.001299752 | 0.01498402 |
| MON1B | 1.15392033 | 0.00454457 | 0.0373485 |
| RET | 1.15586933 | 0.000255544 | 0.00472188 |
| FARP1 | 1.158575 | 1.83×10^-5^ | 0.00069848 |
| COBLL1 | 1.15991678 | 6.11×10^-5^ | 0.00166724 |
| AMIGO3 | 1.16520322 | 1.06×10^-5^ | 0.00048042 |
| TBX1 | 1.16675567 | 0.00335475 | 0.02977856 |
| TXLNGY | 1.17471056 | 6.14×10^-5^ | 0.00167213 |
| SLC43A1 | 1.17716522 | 1.74×10^-7^ | 2.67×10^-5^ |
| TMED3 | 1.17785044 | 5.27×10^-6^ | 0.00030722 |
| FLJ40288 | 1.17917 | 0.001790743 | 0.01899054 |
| DGKK | 1.18061889 | 0.000585828 | 0.00862089 |
| SBK1 | 1.18127667 | 4.67×10^-6^ | 0.00028722 |
| MCF2L | 1.181976 | 0.000306247 | 0.00537267 |
| EPB41L4B | 1.18477889 | 0.00094646 | 0.01202647 |
| AK056098 | 1.18661 | 0.001070664 | 0.01308 |
| L1CAM | 1.20206489 | 0.003480818 | 0.03048586 |
| FRAT1 | 1.20620011 | 1.56×10^-5^ | 0.0006261 |
| MCF2L-AS1 | 1.20997311 | 8.55×10^-5^ | 0.00213108 |
| ELAVL2 | 1.21008778 | 0.005221431 | 0.04114218 |
| MKI67 | 1.21874389 | 0.003329819 | 0.02964983 |
| POSTN | 1.22138222 | 0.000468389 | 0.00733849 |
| MAG | 1.22290967 | 0.001609126 | 0.01759516 |
| CSGALNACT1 | 1.22869333 | 0.000337119 | 0.00574897 |
| LOC100507472 | 1.22970233 | 1.95×10^-5^ | 0.00073117 |
| FZD8 | 1.23434189 | 2.24×10^-5^ | 0.00080678 |
| LOC283177 | 1.24269667 | 0.00028096 | 0.00503675 |
| EPHA5 | 1.24400022 | 0.003262793 | 0.02921823 |
| UAP1 | 1.24759989 | 5.53×10^-5^ | 0.00154918 |
| ST6GALNAC1 | 1.25062545 | 0.001662052 | 0.01798652 |
| ZNF577 | 1.25311978 | 0.000820405 | 0.01090123 |
| RNF157 | 1.25896011 | 0.000940745 | 0.01196189 |
| AFMID | 1.25970444 | 0.000901314 | 0.0116726 |
| NUP210 | 1.26143289 | 4.69×10^-9^ | 2.61×10^-6^ |
| EP300-AS1 | 1.26260056 | 0.002425582 | 0.02355158 |
| RAP1GAP2 | 1.26555711 | 1.76×10^-5^ | 0.00068245 |
| KRTAP11-1 | 1.27398778 | 0.001092169 | 0.01326255 |
| AASDHPPT | 1.276952 | 0.003358461 | 0.02977856 |
| MIR3658 | 1.279574 | 1.00×10^-7^ | 1.74×10^-5^ |
| CENPN | 1.280231 | 0.000873285 | 0.01144246 |
| MIR612 | 1.28187267 | 0.005733957 | 0.04413907 |
| ARHGAP27 | 1.28573322 | 0.000400001 | 0.00651553 |
| MMP26 | 1.28623311 | 5.37×10^-5^ | 0.00150682 |
| ASPHD1 | 1.28672156 | 0.001068155 | 0.01308 |
| ADRB1 | 1.28988011 | 2.52×10^-5^ | 0.00087541 |
| RAB3B | 1.29153745 | 0.00181661 | 0.01921884 |
| TBC1D30 | 1.29372145 | 4.98×10^-5^ | 0.00144863 |
| NEK5 | 1.30054578 | 0.002974511 | 0.02726996 |
| ERG | 1.30066067 | 0.004551248 | 0.0373485 |
| IL31RA | 1.30873489 | 0.006116742 | 0.04630442 |
| DNAH1 | 1.31315922 | 0.002043761 | 0.02081772 |
| PDLIM5 | 1.31626844 | 3.45×10^-5^ | 0.001116 |
| ZNHIT2 | 1.32249578 | 0.001285597 | 0.01488441 |
| CYTH2 | 1.32410667 | 7.18×10^-7^ | 7.29×10^-5^ |
| KCNN4 | 1.32629689 | 0.002316782 | 0.02275239 |
| FAM84A | 1.33002056 | 1.32×10^-5^ | 0.00055434 |
| CBLN1 | 1.33033456 | 0.001306831 | 0.01504728 |
| NPBWR1 | 1.34517411 | 0.001213095 | 0.01426349 |
| TSC22D1 | 1.34912811 | 6.25×10^-5^ | 0.00168298 |
| SAMD5 | 1.35975022 | 0.001036733 | 0.01282598 |
| LOC100996455 | 1.36044245 | 0.006184154 | 0.04662793 |
| MYRIP | 1.36118911 | 0.000510656 | 0.00779901 |
| ZNF765 | 1.36974567 | 3.28×10^-5^ | 0.00107505 |
| GMDS | 1.37207089 | 1.55×10^-8^ | 4.83×10^-6^ |
| OR7E14P | 1.37984667 | 5.97×10^-6^ | 0.00033855 |
| NOV | 1.39411989 | 0.005718774 | 0.04408659 |
| HIST1H3A | 1.39439811 | 0.000667979 | 0.00944953 |
| STMN1 | 1.39512878 | 0.000968412 | 0.01221506 |
| CHDH | 1.39770144 | 5.28×10^-5^ | 0.00149025 |
| DPP4 | 1.410406 | 0.004717445 | 0.03833812 |
| ELL3 | 1.41936167 | 6.84×10^-9^ | 3.08×10^-6^ |
| ATP11B | 1.42862444 | 2.30×10^-5^ | 0.00082042 |
| APOF | 1.45164033 | 4.31×10^-6^ | 0.00027217 |
| PROC | 1.45644656 | 0.000674436 | 0.00951006 |
| GABRB3 | 1.4753 | 0.001532197 | 0.01694024 |
| IAPP | 1.48070089 | 0.004901135 | 0.03945667 |
| FBP1 | 1.48099822 | 1.57×10^-7^ | 2.44×10^-5^ |
| TMC5 | 1.48312411 | 0.001479911 | 0.01657553 |
| MYO6 | 1.49086078 | 9.41×10^-5^ | 0.00229466 |
| INSM1 | 1.49313378 | 0.002652413 | 0.02504144 |
| GJB6 | 1.49988333 | 0.000163569 | 0.00337814 |
| KCNE1 | 1.49996011 | 0.004656551 | 0.03795752 |
| RGS17 | 1.50045789 | 0.001788147 | 0.01899054 |
| LOC101060264 | 1.50180767 | 0.000100959 | 0.00240861 |
| RAB17 | 1.50242645 | 1.52×10^-8^ | 4.83×10^-6^ |
| TMEM238 | 1.50551411 | 0.000938644 | 0.01195126 |
| PLA2G7 | 1.51228578 | 0.00261383 | 0.02480578 |
| KCNN2 | 1.51462344 | 0.001832849 | 0.01932572 |
| GHRHR | 1.51477722 | 0.000737125 | 0.01013268 |
| CECR2 | 1.51881856 | 5.02×10^-5^ | 0.00145575 |
| LOC100996425 | 1.52024022 | 0.004151406 | 0.03489022 |
| TMTC4 | 1.53445522 | 1.60×10^-5^ | 0.00063924 |
| CHRM3 | 1.543605 | 0.002626074 | 0.02485956 |
| CALCA | 1.55602567 | 0.00266033 | 0.02509569 |
| SNX5 | 1.56363044 | 0.005550718 | 0.04308548 |
| AMACR | 1.56541244 | 0.00259247 | 0.02462676 |
| IRX4 | 1.57255189 | 0.000556695 | 0.00830025 |
| DNAH5 | 1.58937622 | 6.13×10^-6^ | 0.00034482 |
| PLA1A | 1.635201 | 0.000105617 | 0.00248833 |
| LOC284930 | 1.63583111 | 0.002314002 | 0.02275239 |
| CTB-167B5.2 | 1.67001311 | 0.000806176 | 0.0107804 |
| RP11-443B7.1 | 1.67104267 | 0.000396453 | 0.00646331 |
| KRT8P17 | 1.69439967 | 0.000256198 | 0.00472741 |
| LOC145837 | 1.71533633 | 0.000390964 | 0.006398 |
| TRPM8 | 1.72273656 | 0.000387651 | 0.00636928 |
| PPM1E | 1.72777356 | 0.000270337 | 0.00490683 |
| ASPN | 1.73214822 | 2.56×10^-6^ | 0.00019411 |
| COL9A2 | 1.73684078 | 1.10×10^-5^ | 0.00049652 |
| LOC101928551 | 1.73734778 | 0.000571051 | 0.00846275 |
| PART1 | 1.74084833 | 8.62×10^-7^ | 8.35×10^-5^ |
| SPAG11A | 1.75286822 | 0.000153115 | 0.00321814 |
| GCNT1 | 1.75650567 | 9.08×10^-5^ | 0.00222615 |
| MS4A8 | 1.760947 | 6.56×10^-6^ | 0.00035716 |
| VSTM2L | 1.76901967 | 8.02×10^-6^ | 0.00040218 |
| LOC101929036 | 1.775728 | 0.001154896 | 0.01373302 |
| MUC3B | 1.77592533 | 0.000142567 | 0.00305766 |
| LOC101928968 | 1.78460667 | 0.000961641 | 0.01215398 |
| STEAP4 | 1.78689867 | 8.44×10^-5^ | 0.00212092 |
| HAGLR | 1.79771378 | 0.000529955 | 0.00801079 |
| HMGA2 | 1.80169422 | 0.00086814 | 0.01138342 |
| FLJ26850 | 1.80847522 | 0.000165506 | 0.00340658 |
| LOC389332 | 1.81851467 | 0.000680727 | 0.00958053 |
| C1orf53 | 1.83913511 | 0.000471813 | 0.0073799 |
| MS4A3 | 1.86588111 | 0.000257573 | 0.00474814 |
| KCNC2 | 1.91139 | 0.002366624 | 0.02315034 |
| GLYATL1 | 1.93799933 | 0.000135452 | 0.00294857 |
| COL2A1 | 1.93811522 | 0.003741857 | 0.03224004 |
| ALDH3B2 | 1.94712633 | 1.56×10^-5^ | 0.0006261 |
| STX19 | 1.96136889 | 1.40×10^-5^ | 0.00058088 |
| TMEM178A | 1.98085711 | 4.75×10^-5^ | 0.0014031 |
| GAL | 2.00140956 | 3.28×10^-7^ | 4.22×10^-5^ |
| PLA2G2A | 2.07693056 | 7.01×10^-6^ | 0.00036886 |
| CRISP2 | 2.10421356 | 0.003088463 | 0.02804253 |
| ERVH48-1 | 2.13626611 | 0.001679508 | 0.01813389 |
| NKX2-3 | 2.16590556 | 0.003393808 | 0.02996542 |
| CBLC | 2.20118789 | 1.27×10^-7^ | 2.13×10^-5^ |
| TARP | 2.25284822 | 5.03×10^-6^ | 0.00029784 |
| ZIC2 | 2.32745867 | 0.002976707 | 0.02727686 |
| GDF15 | 2.333898 | 3.12×10^-6^ | 0.00021529 |
| DLX2 | 2.34586333 | 0.001999068 | 0.02048483 |
| FOLH1 | 2.40042278 | 0.000107431 | 0.00251537 |
| TGM3 | 2.42586245 | 1.44×10^-5^ | 0.00059092 |
| HOXC6 | 2.55551089 | 6.74×10^-5^ | 0.00178015 |
| PCA3 | 2.833002 | 0.000115221 | 0.00261796 |
| FFAR2 | 2.96579867 | 3.72×10^-7^ | 4.57×10^-5^ |
| DLX1 | 3.26100511 | 1.76×10^-5^ | 0.00068245 |

Fold change: Tumor v.s. Normal
