# Supplementary material for: LncRNA CCAT1 promotes prostate cancer cells proliferation, migration, and invasion through regulation of miR-490-3p/FRAT1 axis
Source: Aging (Albany NY). 2021 Jul 28;13(14):18527–44. doi: 10.18632/aging.203300 (PMC8351697; doi:10.18632/aging.203300)
Supplement: Supplementary Tables 3 and 4 [file aging-13-203300-s003.pdf]

Supplementary Table 3. Primer sequences used for qRT-PCR.

Gene symbol	Forward primer (5'-3')	Reverse primer (5'-3')
CCAT1	GCCGTGTTAAGCATTGCGAA	AGAGTAGTGCCTGGCCTAGA
FRAT1	CCACGCCCTGTCTAAAGTGT	TGCAAAACACTGCGCTCTTC
miR-490-3p	CAACCTGGAGGACTCCATGC	
GAPDH	GGAGCGAGATCCCTCCAAAAT	GGCTGTTGTCATACTTCTCATGG
U6	CTCGCTTCGGCAGCACA	AACGCTTCACGAATTTGCGT

Supplementary Table 4. Sequence information for cell transfection.

Gene	Sequence
si-CCAT1	GCAATGCCCTGTTAAGTAA
si-FRAT1	GCTAGTGCTCTCTGGAAAC
miR-490-3p mimics	CAACCUGGAGGACUCCAUGCUG
miR-490-3p inhibitor	CAGCAUGGAGUCCUCCAGGUUG
